# 3D Habitat Complexity and Coral Morphology Modulate Reef Fish Functional Structure in a Marine National Park

**DOI:** 10.1002/ece3.71992

**Published:** 2025-09-01

**Authors:** Sofia B. Ferreira, John H. R. Burns, Atsuko Fukunaga, Lillian J. Tuttle Raz, Sheila A. McKenna, Kailea Annandale, Ryan J. Monello

**Affiliations:** ^1^ Marine Science and Tropical Conservation Biology and Environmental Science, College of Natural and Health Sciences University of Hawaiʻi at Hilo Hilo Hawaii USA; ^2^ Cooperative Institute for Marine and Atmospheric Research University of Hawaiʻi at Mānoa Honolulu Hawaii USA; ^3^ US Geological Survey, Hawaiʻi Cooperative Fishery Research Unit University of Hawaiʻi at Hilo Hilo Hawaii USA; ^4^ National Park Service Pacific Island Inventory and Monitoring Network Volcano Hawaii USA; ^5^ National Park Service Kaloko‐Honokōhau NHP Kailua Kona Hawaii USA

**Keywords:** coral reef ecology, Hawaii, photogrammetry, species assemblage, trait‐based ecology

## Abstract

The ongoing degradation of coral reef habitats is widely acknowledged to have adverse effects on the abundance and diversity of reef fish populations, yet the direct effects on ecosystem functions remain uncertain. This study used a quantitative approach to determine the mechanistic links between fish assemblages and ecological function. We investigated the effects of 3D habitat structure and coral morphology on the ecological, behavioral, and morphological functional traits of reef fish within a protected marine national park. Fish traits such as Gregariousness, Water Column Position, and Body Shape were identified to be highly influential in shaping the multidimensional fish functional space, which was categorized into 10 Fish Functional Groups (FFG). Furthermore, habitat complexity and coral morphology significantly explained the abundances of eight out of 10 FFG. Notably, the habitat complexity metrics of Slope and Surface Complexity, along with coral morphologies of Branching and Mounding types, emerged as the most influential habitat features across FFG. Pairing Compressiform species and Schooling Short/Deep species, for example, significantly increased in abundance on substrate with higher Slopes and increased percentages of branching coral cover. Additionally, Cryptic and Nocturnal species exhibited statistically significant associations with all coral morphologies and substrates with high trait values of Slope and Curvature. Elucidating ecological drivers of specific functional groups of reef fish is critical for determining how changes in reef composition and structure will alter fish assemblages. Broad scale patterns were also detected, suggesting that although structural complexity is important, live coral morphologies have a greater positive impact on reef fish functional groups. These findings have direct implications for conservation and monitoring efforts, offering valuable insights for predicting the impacts of environmental change on community dynamics and ecosystem functioning.

## Graphical abstract

This study investigates how 3D habitat complexity and coral morphology shape reef fish functional groups. We found that both coral morphologies, particularly branching and mounding corals, and structural complexity metrics like slope and vector terrain ruggedness significantly drive fish functional group abundance. However, coral morphologies emerged as the more influential predictor, underscoring the importance of preserving coral morphological diversity for maintaining functional fish communities, with direct implications for conservation efforts.

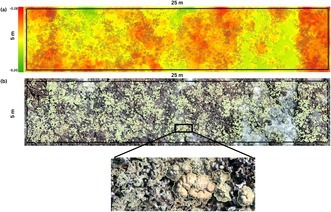

## 1 Introduction

It is widely recognized that the physical structure of a habitat significantly impacts biodiversity and ecological functioning (Brose and Hillebrand 2016; Kovalenko et al. 2012). In coral reef ecosystems, Scleractinia corals function as ecosystem engineers of structural complexity by secreting calcium carbonate skeletons with intricate morphological variations (Jones et al. 1994; Guendulain‐Garcia et al. 2024; Wang et al. 2021). The underlying reef topography provides additional abiogenic structures such as rocks, boulders, spur and groove formations, and sloping continental or islandic shelves (Asbury et al. 2023; Zieger et al. 2009). The high structural heterogeneity provided by these biotic and abiotic features is linked to the outstanding biodiversity and productivity of coral reef ecosystems, as higher complexity provides spatial refuges from predation, creates niche spaces, increases food availability, and supplies nesting sites (Bozec et al. 2015; Ebeling and Hixon 1991; Robertson and Sheldon 1979). Examining how specific metrics of 3D reef complexity impact the functional structure of reef fish can deepen our understanding of the connections between habitat dynamics and ecological processes.

The structure of reef fish assemblages is closely associated with the availability of suitable refuge spaces and foraging opportunities (Catano et al. 2016; Hixon and Beets 1993). This association often produces a strong positive relationship between complex habitats with ample live coral cover and reef fish abundance and diversity (Agudo‐Adriani et al. 2019; Bell and Galzin 1984; Coker et al. 2014; Darling et al. 2017; Komyakova et al. 2013). While the influence of habitat complexity on fish species assemblages has been extensively studied, there is now a growing emphasis on understanding reef fish assemblages in terms of their functional roles within ecosystems, rather than solely based on taxonomic identity (Kiørboe et al. 2018; Luiz et al. 2019). This paradigm shift is prompted by unprecedented rates of coral mortality and subsequent loss of reef structural complexity, leading to cascading loss of reef fish biomass and diversity, as well as their associated ecological functions and services (Alvarez‐Filip et al. 2009; Pratchett et al. 2018; Morais et al. 2020). Enhancing our understanding of the connections between functional fish traits, coral reef community composition, and habitat complexity will aid conservation efforts to protect and sustain reef features that drive key ecological functions.

Reef fish populations play integral roles in coral reef ecosystems, contributing to crucial processes, such as herbivory, bioerosion, trophic stability, and nutrient cycling (Brandl et al. 2019; Schiettekatte et al. 2022). Effective management of transitioning reefs requires an understanding of reef fish assemblage structure that extends beyond their taxonomic identities to encompass their vital ecological roles. Trait‐based ecology offers a valuable approach to comprehending the assemblage structure and its impact on ecosystem functions (Green et al. 2022; Kiørboe et al. 2018; Kremer et al. 2017; Mason and De Bello 2013). Traits used to characterize an organism can encompass inherited morphological, behavioral, and physiological characteristics that shape a species' role within a reef community and its relationship with ecosystem functions (McGill et al. 2006; Violle et al. 2007). Reef fish exhibit a diverse array of traits that influence their ecological roles in the reef ecosystem (Hadj‐Hammou et al. 2021; McLean et al. 2021). For example, cryptic behavior can significantly impact predator–prey interactions (Depczynski and Bellwood 2004), whereas traits such as mobility and gregariousness can affect nutrient cycling rates (White and Warner 2007; Williams et al. 2018). Owing to their ability to simultaneously address assemblage structure and ecosystem functioning, trait‐based functional approaches have attracted considerable attention in coral reef ecology (Hodge and Price 2022; Madin et al. 2016; Pombo‐Ayora et al. 2020; Villéger et al. 2017). While more studies in this field are adopting a trait‐based approach, additional research can help identify which traits respond to changes in coral composition and habitat complexity.

Reef fish display various levels of reliance on habitat complexity, ranging from highly specialized species that depend critically on specific coral species for food or shelter to those with weaker associations with live corals and underlying substrate types (Pratchett et al. 2012; Wilson et al. 2008). Specialized species often exhibit selectivity towards particular coral morphologies, such as branching or tabling (Kerry and Bellwood 2012; Untersteggaber et al. 2014), and structural complexity characterized by the metrics of reef rugosity and terrain ruggedness (Fukunaga and Burns 2020; Ménard et al. 2012). Although these preferences have been considerably observed through species‐based analyses, investigating these associations using trait‐based approaches may shed additional light on the underlying ecological mechanisms driving these preferences. For instance, behavioral traits, such as crypsis or gregariousness, may explain why less social and cryptic species are more reliant on habitat complexity than fish in large schools (Depczynski and Bellwood 2004; Gardiner and Jones 2010). Similarly, the water column position of fish shows that bottom‐dweller species exhibit stronger relationships with reef benthic complexity than do species swimming higher in the water column (Urbina‐Barreto et al. 2022), and morphological traits such as body size may influence reef fish associations with fine‐scale habitat complexity (Alvarez‐Filip et al. 2011). Given the links between fish traits and ecological functions, these insights can aid in understanding broader ecological dynamics, which can then aid in conservation efforts.

Significant knowledge gaps exist regarding the selection and combination of fish traits that should be associated with habitat characteristics of coral reefs; thus, there has been an inconsistent use of trait‐based applications in the coral reef ecology literature (Anderson et al. 2022; Darling et al. 2017; Helder et al. 2022; Richardson et al. 2017; Urbina‐Barreto et al. 2022). For example, while trophic groups are commonly used to categorize reef fish when examining fish‐habitat relationships (Agudo‐Adriani et al. 2019; Fukunaga et al. 2020), there is a notable lack of attention to the broader spectrum of fish behavior and morphological traits. Theoretical frameworks, however, suggest that species assemblage may be better explained through a combination of traits rather than isolated traits (Verberk et al. 2013). There has also been a limited consideration of coral metrics beyond coral cover percentage, overlooking the pivotal role of coral morphology in providing differential forms of structural complexity to the reef habitat (Burns et al. 2019; Ferreira et al. 2023; Zawada et al. 2019). The incorporation of advanced technologies such as Structure from Motion (SfM) photogrammetry offers a unique opportunity for more comprehensive and precise quantification of habitat features, with significant potential to deepen our insight into the influence of habitat complexity on reef fish assemblages (Fukunaga et al. 2020; Helder et al. 2022; Urbina‐Barreto et al. 2021). Conducting a comprehensive trait‐based approach and examining associations with detailed measures of coral reef communities and habitat complexity can clarify which measures are best suited to enhance our understanding of coral reef ecology.

Our study aims to address these gaps by pursuing the following objectives: (1) characterize the functional structure of fish assemblages within a marine national park, incorporating a range of trophic, behavioral, and morphological fish traits; (2) evaluate the utility of photogrammetry‐derived measures of coral morphology and structural complexity metrics in explaining the functional assemblage of fish; and (3) determine the relative significance of coral traits compared to habitat‐wide structural complexity in shaping the functional structure of reef fish.

## 2 Materials and Methods

### 2.1 Study Site

**FIGURE 1:**
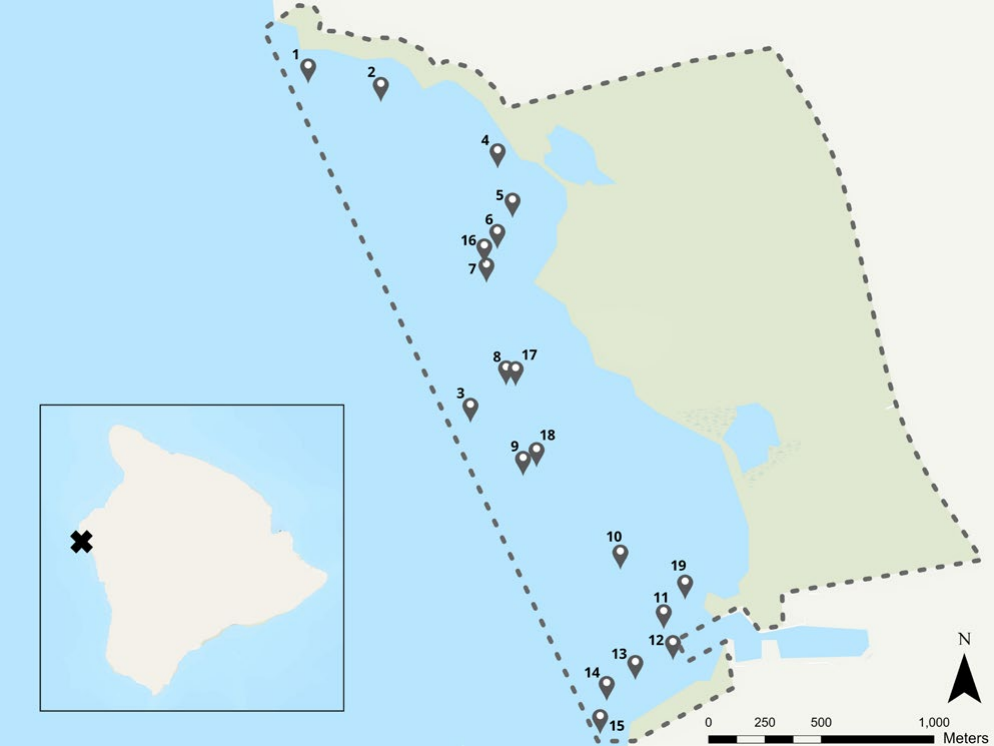
Map of the study site in Kailua‐Kona, Hawaii, USA. The gray dashed line represents the Kaloko‐Honokohau National Historical Park (KAHO, 19° 40′41.9808″ N, 156° 1′13.7172″ W) boundary.

The study site is adjacent to Kailua‐Kona, Hawaii, within the Kaloko‐Honokohau National Historical Park (KAHO, 19°40′41.9808″ N, 156°1′13.7172″ W, Figure 1). KAHO is one of four National Park lands located along the western coast of the Island of Hawaii and the only one that includes submerged lands and marine resources within its official boundaries. Established in 1978, the park spans 598 submerged acres and benefits from protection granted by various legislative acts, including the National Park Service Organic Act (1916), National Park System Resource Protection Act (16 U.S.C. § 19jj), and Redwoods Act (1978). The KAHO submerged lands and marine resources have been routinely monitored by the National Park Service Pacific Island Inventory and Monitoring Network since 2000 (Brown et al. 2016). The study area exhibits stable salinity, low turbidity, and moderate wave exposure, with southern sites more exposed to North Pacific swell (figure 1; Raikow et al. 2021). While Submarine Groundwater Discharge is a prominent feature of the nearshore environment, bottom water quality conditions remain relatively homogeneous across sites (Raikow et al. 2021). For this study, we surveyed 19 study plots ranging from 10 to 20 m in depth, each measuring 25 × 5 m, within the park boundaries between November 2021 and April 2022. Fifteen of these plots were part of the National Park Service's (NPS) annual monitoring, while the remaining four were added in for this study and surveyed within the same timeframe.

### 2.2 Fish Surveys and Trait Characterization

Reef fish assemblages were assessed using an underwater visual census (UVC) following the standardized National Park Service (NPS) Pacific Island Network monitoring protocols (Brown et al. 2011). At each of the 19 study plots, SCUBA divers laid a 25‐m transect parallel to the reef crest along a constant depth. Surveys were conducted by three trained and experienced observers, who followed consistent protocols to reduce variability. At each site, observers waited briefly to allow fish to acclimate and swam slowly and minimally through the transect area, recording all fishes observed within 2.5 m on either side across the entire water column from seafloor to surface. Fish trait data, including trophic group, gregariousness, crypsis, mobility, water column position, activity period, maximum body length, and body shape, were obtained from the literature and FishBase (Froese and Pauly 2014). These traits were chosen based on their direct relevance to ecological functions within coral reef ecosystems that have been described in the literature (Table 1). See Data S1 for more detail on trait characterization methodologies.

**TABLE 1:** Descriptions and ecological importance of the fish traits used in this study.

Type	Trait	Trait values	Ecological importance
Ecological	Trophic group	Planktivore, Omnivore, Invertivore, Corallivore, Piscivore, Browser, Excavator/Scraper, Grazer/Detritivore	Mediates ecological niche, nutrient cycling, and trophic interactions (Cantor et al. 2018; Van Wert et al. 2023)
Morphological	Max body size	Continuous numerical	Mediates ecological niche, energy needs, and predation or fishing vulnerability (Fisher et al. 2010; Graham et al. 2005; Kulbicki et al. 2015)
	Body shape	Compressiform, elongated, fusiform, short/deep	Mediates predator–prey interactions and resource utilization (Green and Côté 2014)
Behavioral	Crypsis	1 (not‐cryptic), 2 (cryptic)	Mediates predator–prey interactions and resource utilization (Brandl et al. 2018; Depczynski and Bellwood 2004)
	Mobility	Sedentary, mobile, wide	Mediates fish energy needs, nutrient transfer, and predator–prey interactions (Caldwell and Gergel 2013)
	Gregariousness	Solitary, pairing, schooling	Mediates ecological niche, predator avoidance or vulnerability, resource utilization (Gardiner and Jones 2010; Shantz et al. 2015; White and Warner 2007)
	Water column position	Bottom, low, high	Mediates niche partitioning (Fulton et al. 2001)
	Activity period	Diurnal, nocturnal	Mediates predation susceptibility, predator–prey interactions, and resource utilization (Collins et al. 2022; McCauley et al. 2012)

### 2.3 Photogrammetry Surveys

Following the fish surveys, all study plots were surveyed using structure‐from‐motion (SfM) photogrammetry techniques. Four scale bars were positioned 2.5 m from each side and each end of the 25 m transect to define the plot area (125 m^2^) and to serve as ground control points (GCPs) for accurate spatial orthorectification of the 3D models and resulting data products (Burns et al. 2015). The SCUBA diver then systematically swam over the plot area, capturing continuous and overlapping (70%–80%) images of the reef substratum using a single‐lens reflex (SLR) camera. To ensure full image alignment and model accuracy, divers photographed an area approximately 1–2 m beyond the 25 × 5 m plot boundaries, allowing the final 3D reconstruction to retain high resolution within the target survey area. These images served as input data for generating 3D reconstructions of the reef plots using the Agisoft Metashape software v.1.7.1 (Agisoft LLC, St. Petersburg, Russia). Following the methodology outlined by Burns et al. (2015), this process involved aligning images, generating sparse and dense point clouds, constructing polygon mesh models, overlaying textures, and rendering both Digital Elevation Models (DEMs) and high‐resolution two‐dimensional (2D) orthomosaics from an overhead perspective.

### 2.4 Coral Digitization

**FIGURE 2:**
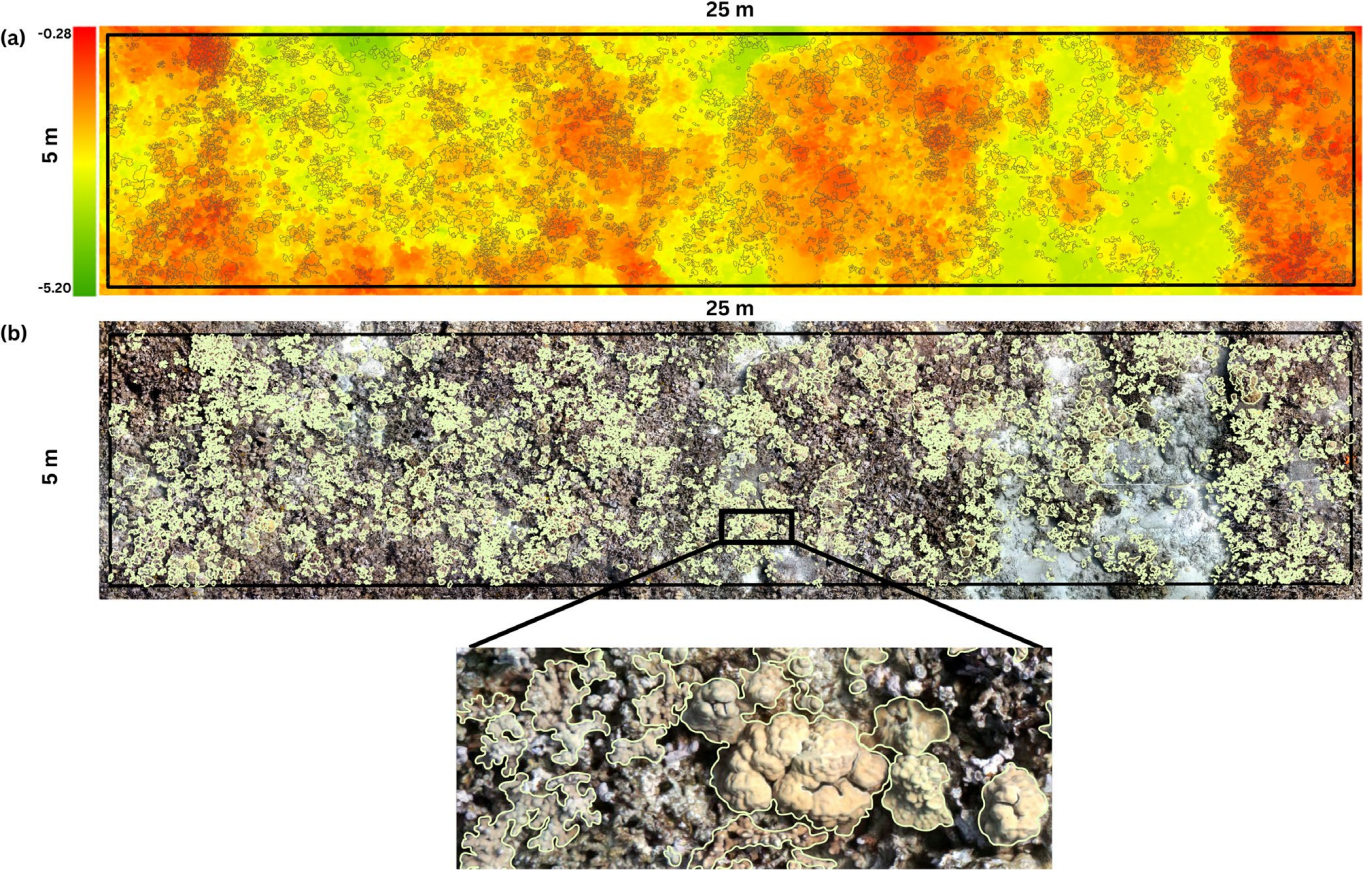
An example of a 25 × 5 m study plot with 3700 annotated coral colonies. (a) Digital Elevation Model (DEM) with overlaid coral polygons and (b) Orthomosaic with overlaid coral polygons.

Using the geospatial software ArcGIS (ArcGIS 10.8, Environmental Systems Resource Institute, Redlands, USA), each full DEM and orthomosaic was imported into GIS, and a 25 × 5 m polygon was overlaid to delineate the survey area and ensure uniform plot sizes for analysis. Subsequently, every coral colony within the 125‐m^2^ plot area was digitized manually as polygons by tracing the edges of the individual colonies (Figure 2). Colonies were only surveyed if at least 50% of their area fell within the boundaries of the 25 × 5 m plot polygon. Each colony was identified at the species level and assigned a morphological category following the definitions and naming conventions outlined in table 2 of Winston et al. (2020). Geospatial analyses were conducted in R (version 4.3.0) using the raster, sf, and exactextractr packages (R Core Team 2021; Hijmans 2025; Pebesma 2018; Baston 2022). For each colony, we calculated the total three‐dimensional surface area by integrating the digitized polygon boundaries with the spatially aligned digital elevation model derived from the same photogrammetric model (Jenness 2004; House et al. 2018). Custom R scripts were used to extract elevation values within each polygon and compute surface area based on topographic variation. These values represent true 3D surface area, from which the mean colony area per plot was calculated for further analyses. To calculate the coral morphology metrics at the level of each study plot, we aggregated the total two‐dimensional cover (% area) for every observed coral morphology by summing the areas of all colonies with each morphology type and dividing it by the total plot area (i.e., 125 m^2^, Table 2). See Data S1 for more detail on morphology characterization methodologies.

**TABLE 2:** Description and graphical representation of habitat complexity metrics and coral trait metrics.

Habitat feature	Metric	Explanation/Calculation	Graphic representation
Complexity	Slope	Measure the steepness at each raster cell. Calculated for each raster cell at 1 cm resolution, and then averaged by study plot.	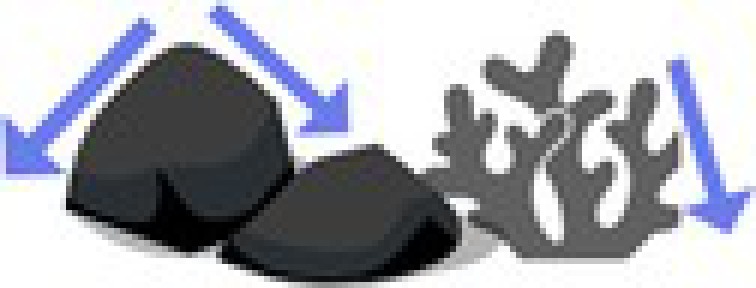
	Vector ruggedness measure (VRM)	Measures the variation in three‐dimensional orientation of raster cells using vector dispersion analysis. Calculated for each raster cell at 1 cm resolution, and then averaged by study plot.	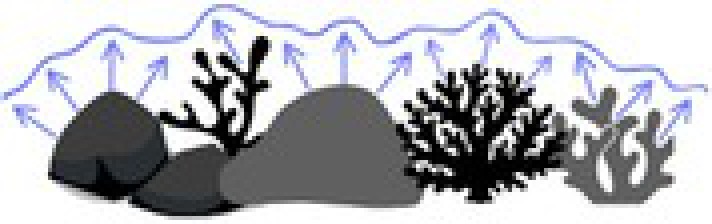
	Curvature	Measures convexity and concavity. Calculated for each raster cell at 1 cm resolution, and then averaged by study plot.	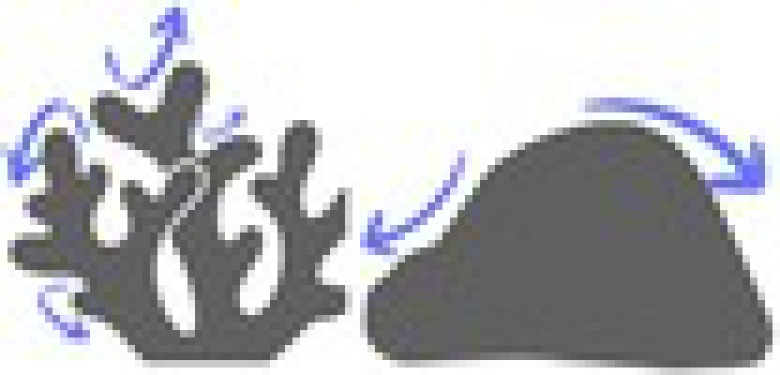
	Surface complexity	Measures ratio of 3D over 2D surface area for the entire DEM. Also known as reef rugosity.	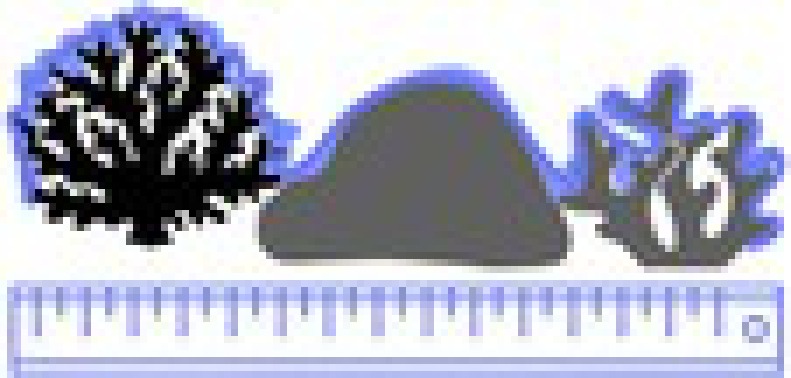
Coral	Morphology	Mounding, Encrusting, Branching, Knobby. Calculated as the total 2D % cover within the 125 m^2^ plot area.	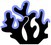
	Size (3D area)	Total three‐dimensional surface area of each individual coral colony, calculated using colony polygon boundaries and elevation data from the spatially aligned digital elevation model (DEM). Calculated for each coral colony and then averaged by plot.	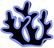

### 2.5 3D Habitat Complexity

Digital Elevation Models (DEMs) rendered from 25 × 5 m study plots were geospatially analyzed in R with raster and sf packages (Hijmans 2023; Pebesma 2018). The cropped DEMs representing the study plots were analyzed using the computational procedures described by Fukunaga et al. (2019) to quantify structural complexity metrics, including surface complexity, slope, vector ruggedness measure (VRM), and profile curvature. Table 2 provides the details of each metric, including how it is computed and the type of reef features captured by these metrics.

### 2.6 Statistical Analysis

To characterize the overall functional structure of the fish assemblage observed in this study, we computed pairwise trait‐based distances between species using the mFD R package and the funct.dist function with the Gower dissimilarity index, known for accommodating ordinal, nominal, and quantitative traits (Gower 1971; Magneville et al. 2022). Principal Coordinate Analysis (PCoA) was then conducted on the Gower matrix to illustrate the multidimensional functional trait space occupied by all species within the assemblage, across all 19 study plots. All statistical analyses were completed using R v.4.3.0 (R Core Team 2024) within RStudio (Posit Team 2025).

To ensure that this functional trait space accurately represents species traits, we assessed the space's relative quality by quantifying how well the PCoA Euclidean‐based functional distance preserved the Gower‐based trait distances. To do so, we calculated the mean absolute deviation (MAD) index via the mFD::quality.fspaces function. The MAD index measures the average deviation between the original Gower distances and the PCoA‐reconstructed distances, with lower MAD values indicating higher quality because the functional space more faithfully reflects the original trait distances (Magneville et al. 2022). After evaluating the space's quality, we retained the optimal number of axes based on the axis number with the lowest MAD value for subsequent analyses.

We then computed the functional diversity indices of Functional Richness (FR), Functional Evenness (FE), and Functional Dispersion (FD) for each plot. These indices quantify the amount of functional trait space occupied by a species assemblage, the regularity of abundance distribution within this space, and the spread of species weighted by their relative abundance, respectively (Cornwell et al. 2006; Laliberté and Legendre 2010; Villéger et al. 2008). The contribution of species traits to functional space was quantified using the mFD::traits.faxes.cor function, which computes the correlation values between traits and PCoA axes. Lastly, hierarchical clustering was performed on the trait‐based Gower distance matrix using the stats::hclust function with Ward's minimum variance method to define fish functional groups based on their trait similarities.

The influence of 3D habitat complexity and coral traits on the overall abundance of individual fish functional groups was determined using generalized linear models (GLMs), utilizing a negative binomial family to address overdispersion in the data. Two sets of models were constructed for each functional group abundance: one incorporating only coral traits as predictor variables and another incorporating habitat complexity. Prior to model fitting, predictor variables were scaled for coefficient comparability. The Stepwise Akaike Information Criterion (AIC) procedure using the MASS package and stepAIC function through backward selection was implemented for model selection (Venables et al. 2002). Model diagnostics including residual plot examination and goodness‐of‐fit statistics and variance inflation factor (VIFs) assessments were conducted to evaluate the selected models. To further explore the effect of habitat characteristics on fish traits, we conducted supplementary negative binomial GLMs for each trait value. These models were also partitioned by coral traits and habitat complexity metrics and subjected to backward AIC‐based stepwise selection. The pseudo‐explained variance of each selected model was calculated by subtracting 1 from the ratio of residual deviance and null deviance (Hardin and Hilbe 2007).

For each of the negative binomial GLMs, we calculated the incidence rate ratios (IRRs) by exponentiating the estimated model coefficients for each predictor variable. The IRRs provide a multiplicative factor by which the abundance of the fish functional group is expected to change for a one‐unit increase in the predictor variable, holding all other variables constant (Hilbe 2011). Specifically, an IRR > 1 indicates that an increase in the predictor variable is associated with an increase in the abundance of the functional group, while an IRR < 1 suggests a decrease. These ratios were visualized using the sjPlot::plot_models function, which automatically computes and displays the IRRs for each model (Lüdecke 2025). Lastly, we conducted a Multivariate Redundancy Analysis (RDA) using the vegan package to examine the relationships between 3D structural complexity metrics (response matrix) and coral morphological group percent cover (predictor matrix). Coral morphology values were untransformed, and all 3D metrics were scaled to standardize units prior to analysis. Multicollinearity was assessed through Variance Inflation Factor (VIF), and model significance was evaluated with a permutation test (999 iterations).

## 3 Results

A total of 77 fish species were surveyed across all the study plots, with species richness ranging from 17 to 38 per plot. Total fish abundance varied considerably, ranging from 51 to 371 individuals per plot and averaging 213 individuals across all plots (Figure S1). Among coral morphologies, Mounding exhibited the highest 2D percentage cover, averaging 5.7% across plots and reaching a maximum of 18.5% at one location. The Encrusting, Branching, and Knobby morphologies followed, with average covers of 2.4%, 1.5%, and 1%, respectively (Table S1).

The highest‐quality fish functional trait space consisted of a four‐dimensional PCoA space (MAD = 0.05, Figure S2). For the convenience of graphical display, only the first two axes are plotted here, representing a cumulative 66.9% of the variance in the data explained by the PCoA model (Figure 4a; additional axes are plotted in Figure S3). The first PCoA axis was most strongly correlated with the fish traits of Gregariousness (*r* = 0.53), Water Column Position (*r* = 0.48), and Mobility (*r* = 0.48); whereas the second PCoA axis was primarily correlated with Body Shape (*r* = 0.79) and Trophic Group (*r* = 0.39; Table S2).

**FIGURE 3:**
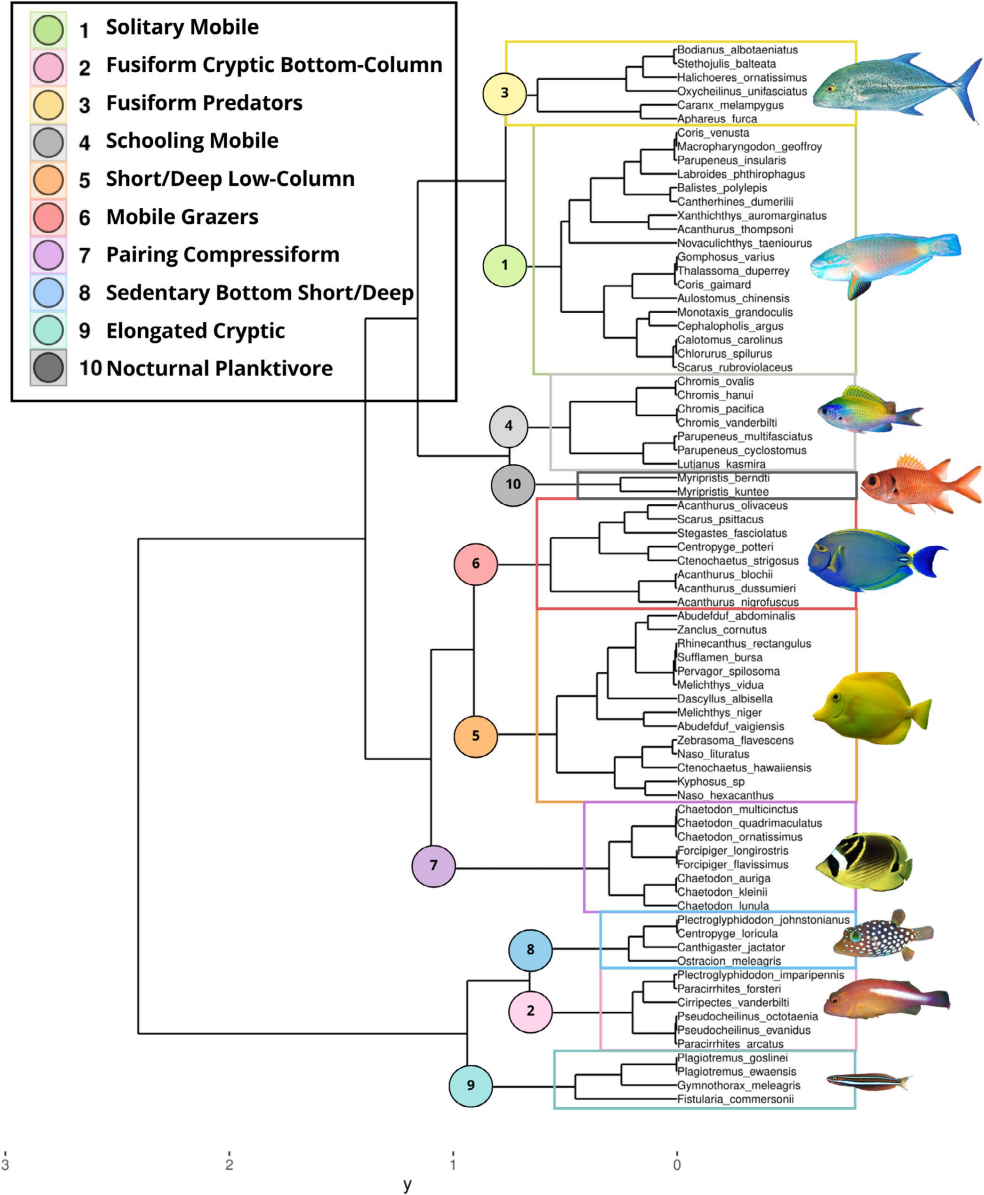
Dendrogram displaying species partitioning across the 10 trait‐based hierarchical clusters.

**FIGURE 4:**
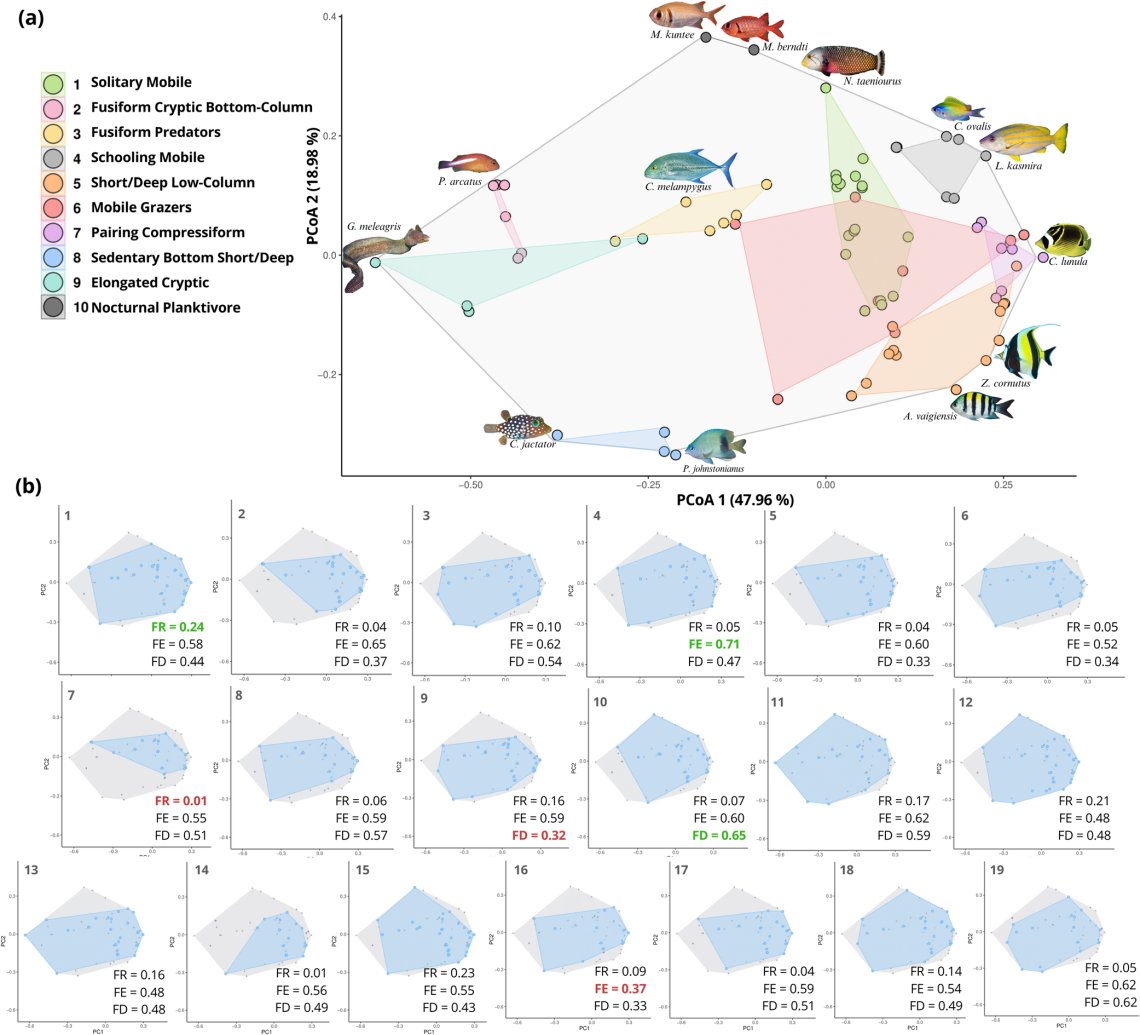
(a) Two‐dimensional functional trait space of 77 fish species based on Gower‐distance Principal Coordinate Analysis (PCoA). The first two axes explained 47.96% and 18.98% of the variability, respectively. Dots represent individual species colored according to hierarchical cluster analysis, with 11 species depicted at the vertices of the convex hull (full species names provided in Table S4). The legend specifies the top two or three traits that differentiate each cluster. Clusters 10 does not display ellipses due to limited species (< 4) within the clusters. (b) Functional richness of each study plot is shown by the volume of the convex hull (blue) overlaid onto the community functional space from Figure 3a above (gray). Plot‐based values for Functional Richness (FR), Evenness (FE), and Dispersion (FD) are indicated, with green and red values denoting the highest and lowest values for each diversity index, respectively.

The hierarchical cluster analysis resulted in 10 distinct clusters, hereafter referred to as Fish Functional Groups (FG, Table 3, Figure 3). Plotted on the functional trait space of the first two PCoA axes, relatively social species that exhibit Schooling or Pairing behavior clustered on the right side of the ordination space (FG 4, 5, 7), whereas solitary species dispersed within the top‐middle and left sides of the ordination space (Figure 4a). Fish were also separated by their water‐column position, with sedentary species living in close association with the bottom clustering towards the left‐ and bottom‐left sides of the functional space (FG 2 and 8) and more mobile species positioned in the right spaces (Figure 4a). Functional Richness (FR) was highest at plot 1 (0.24), in contrast with the lowest value observed at plot 7 (0.01, Figure 4b). Functional Evenness (FE) was highest at plot 4 (0.71) and lowest at plot 16 (0.37, Figure 4b). Lastly, Functional Dispersion (FD) ranged from a value of 0.65 (plot 10) to 0.32 (plot 9, Figure 4b).

**TABLE 3:** Predominant trait values for each of the 10 functional groups.

FG	Trophic group	Body shape	Body size (range)	Mobility	Water column	Gregariousness	Crypsis	Activity period
1	Mix	Mix	12–80 cm	Mobile	Low	Solitary	No	Diurnal
2	Grazer/Detritivore Invertivore Omnivore	Fusiform	6–22 cm	Sedentary	Bottom	Solitary	Yes	Diurnal
3	Invertivore Piscivore	Fusiform	15–117 cm	Mix	Mix	Solitary	No	Diurnal
4	Omnivore Planktivore	Fusiform	6–50 cm	Mobile	Mix	Schooling	No	Diurnal
5	Mix	Short/Deep	13–75 cm	Mobile	Low	Mix	No	Diurnal
6	Grazer/Detritivore	Mix	10–54 cm	Mobile	Low	Mix	No	Diurnal
7	Mix	Compressiform	12–24 cm	Mobile	Low	Pairing	No	Diurnal
8	Omnivore Grazer/Detritivore	Short/Deep	9–25 cm	Sedentary	Bottom	Solitary	Mix	Diurnal
9	Piscivore	Elongated	6–160 cm	Mix	Bottom	Solitary	Yes	Diurnal
10	Planktivore	Fusiform	26‐30 cm	Mobile	Low	Schooling	Yes	Nocturnal

*Note:* “Mix” indicates that the group exhibited more than half of the possible trait categories for a given trait type with no clear predominance. For additional details, refer to Figure S3.

**FIGURE 5:**
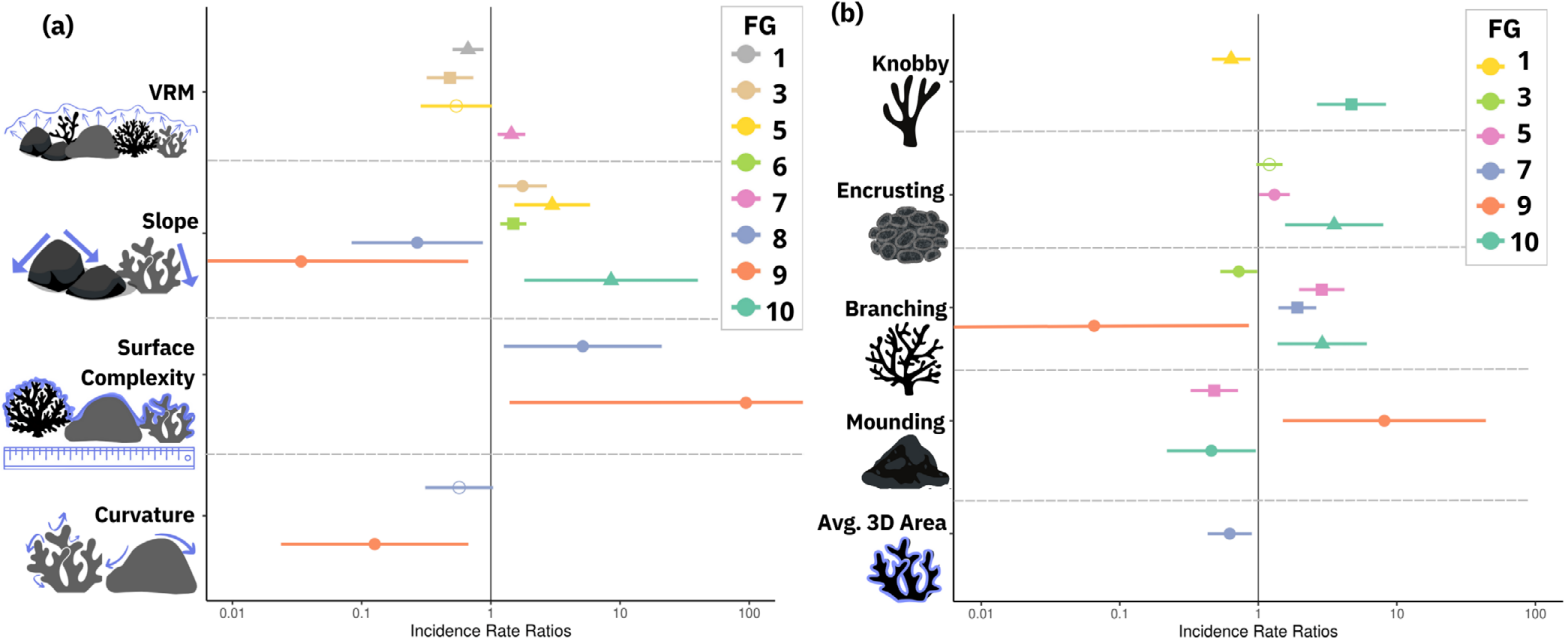
Incidence rate ratios and 95% confidence intervals (CI) derived from negative binomial generalized linear models depicting the relationship between fish functional group abundance and (a) structural complexity and (b) coral morphology. Points are colored by fish Functional Group (FG), and shapes represent statistical significance levels (hollow circle = not significant, filled circle = *p* < 0.05, square = *p* < 0.01, triangle = *p* < 0.001). Functional groups are: FG1 = Solitary Mobile, FG2 = Fusiform Cryptic Bottom‐Column, FG3 = Fusiform Predators, FG4 = Schooling Mobile, FG5 = Short/Deep Low‐Column, FG6 = Mobile Grazers, FG7 = Pairing Compressiform, FG8 = Sedentary Bottom Short/Deep, FG9 = Elongated Cryptic, FG10 = Nocturnal Planktivore. Legend title abbreviation FG = Functional Group.

Both habitat complexity and coral traits exhibited statistically significant relationships with the abundance of 8 out of 10 Functional Groups (FG, Figure 5, Table S3). Fusiform Predators from FG 2 increased in abundance with higher values of Slope yet decreased with higher percentages of Branching corals (FG 3, Figure 5, Table S3). The abundance of Nocturnal species (FG 10) strongly increased with higher coverage of Knobby, Encrusting, and Branching corals, as well as with higher Slope values (Figure 5, Table S3). In contrast, FG1 species, characterized by Solitary and Mobile behavior, exhibited greater abundance as structural complexity, measured by VRM and the percentage of Knobby corals, declined (Figure 5). Meanwhile, Elongated Cryptic species from FG9 showed notable responses to various complexity metrics and coral morphologies, though there was uncertainty in these responses (Figure 5, Table S3).

**FIGURE 6:**
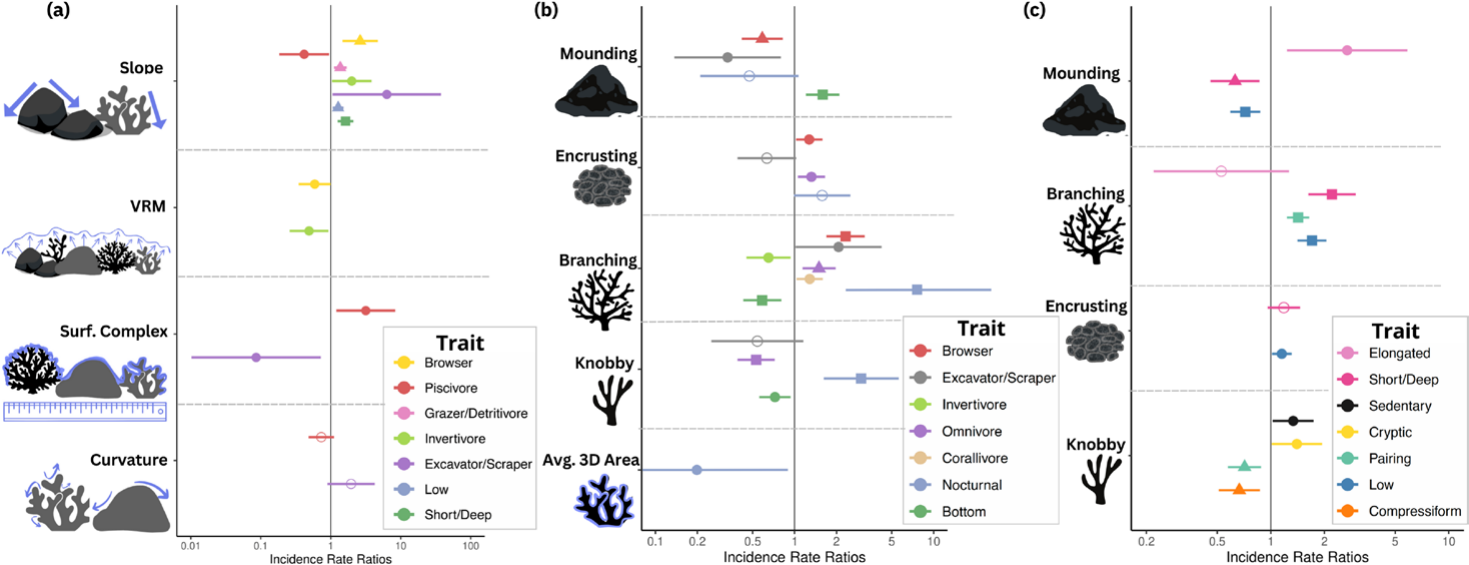
Incidence rate ratios and 95% confidence intervals (CI) derived from negative binomial generalized linear models depicting the relationship between fish abundance by trait and (a) habitat complexity metrics and (b) and (c) coral morphology (split for clearer visualization & equal axes). Points are colored by trait category, and shapes represent statistical significance levels (hollow circle = not significant, filled circle = *p* < 0.05, square = *p* < 0.01, triangle = *p* < 0.001).

Single‐trait analyses showed that trophic groups are highly influenced by habitat features, with five trophic groups significantly related to coral traits and five related to structural complexity. The complexity metric of Slope showed strong positive associations with the abundance of Browsers, Grazer/Detritivores, Invertivores, and Excavator/Scrapers, while increased Surface Complexity showed a positive effect on Piscivores (Figure 6, Table S4). The abundance of Browsers, Excavator/Scrapers, Omnivores, and Corallivores increased with increasing percent cover of Branching corals (Figure 6, Table S4). Conversely, Invertivores decreased in abundance as the percentage cover of Branching corals increased (Figure 6).

**FIGURE 7:**
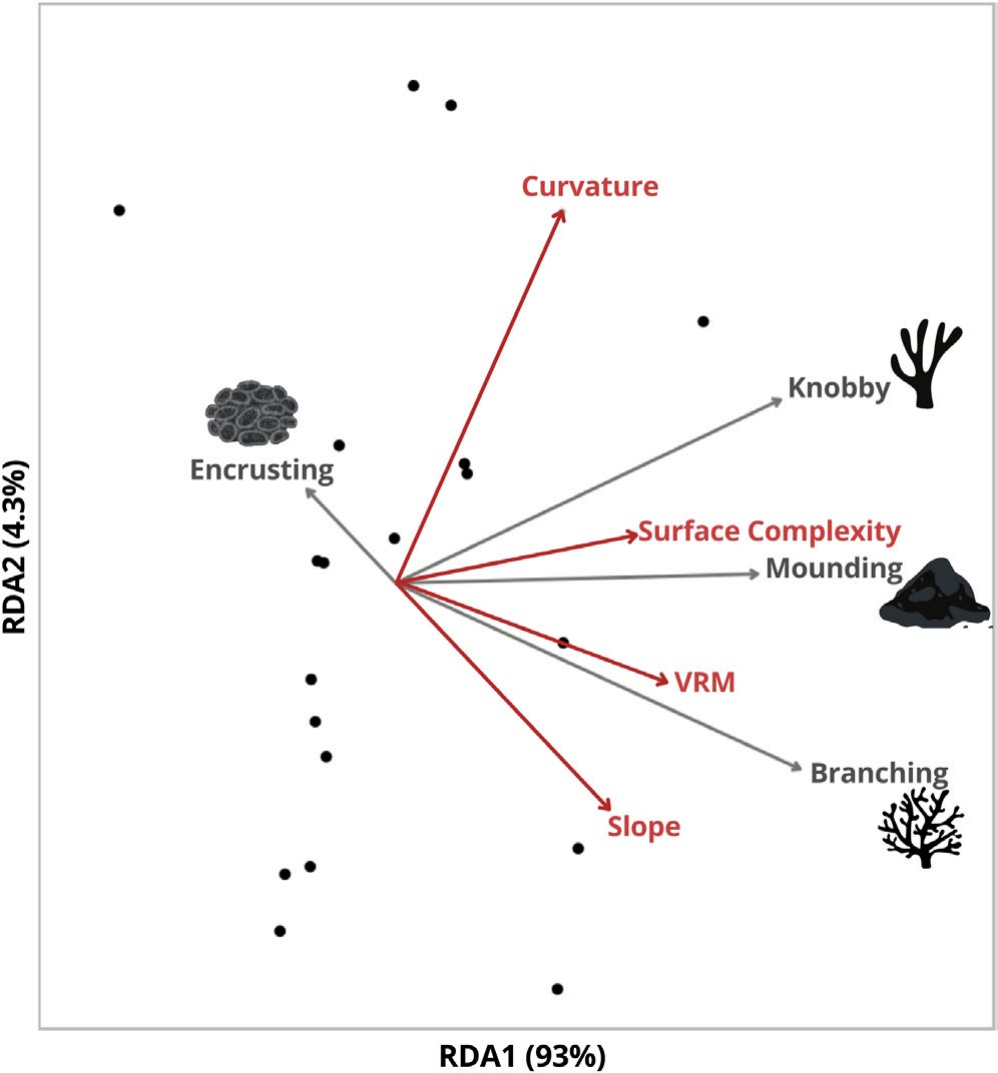
Redundancy Analysis (RDA) biplot showing the relationships among structural complexity metrics (red vectors) and coral morphology (gray vectors). Black dots represent each of the individual study plots (*n* = 19). The first two axes explained 93% and 4.3% of the explained variation within the data, respectively (Adj *R* ^2^ = 0.36, Permutest *p* = 0.01).

The morphological traits of coral colonies showed strong influences on the abundance of behavioral traits. Interestingly, the abundance of fish with Bottom water column behavior showed a negative response to Branching and Knobby corals, yet the abundance of fish within the Low water column group exhibited positive responses to Branching and Encrusting corals, as well as higher Slope values (Figure 6, Table S4). The abundance of fish exhibiting a Short/Deep body form increased with a higher percent cover of Branching corals and Slope values, while Compressiform fish abundance decreased with a higher percentage of Knobby corals, and Elongated increased with Mounding corals (Figure 6, Table S4). Finally, the abundance of cryptic species increased with an increase in Knobby coral cover (Figure 6, Table S4). The Redundancy Analysis (RDA) model accounted for 93% and 4% of the total variation along the first and second axes, respectively (Figure 7). Permutation testing confirmed that the model was statistically significant (*p* = 0.014), with an adjusted *R* ^2^ of 36.2%.

## 4 Discussion

Understanding community structure is one of the first steps towards a deeper understanding of ecosystem functioning, as it offers insights into the relative importance of different groups of species within the ecosystem (McGill et al. 2006). We explored the distribution of eight categories of coral‐reef fish traits within a multidimensional functional space to delineate functional similarities among coexisting species and elucidate their ecological roles. Notably, morpho‐behavioral traits such as gregariousness, water column position, body shape, and body length strongly structured the fish functional space (Figure 4a, Table S2). Similar trait effects have been observed in Indonesia, Brazil, and Australia, where differences in these traits drove fish dispersion in functional space (Carvalho et al. 2021; Reverter et al. 2021; Richardson et al. 2017). This detailed understanding of trait‐based functional space lays the foundation for assessing how ecological factors and functional groupings further influence species distributions and interactions across diverse habitats.

Although species clustering within the functional space may be influenced by shared phylogenetic ancestry and consequent trait similarities (Floeter et al. 2018), ecological linkages may also be significant contributors. For instance, while all *Chaetodon* butterflyfishes clustered within Functional Group (FG) 7, *Acanthurus* surgeonfishes were distributed across FGs 1 and 6, likely reflecting differences in diet and behavior (figure 3, Robertson et al. 1979). Our clustering results also showed notable dispersion among herbivore types, showing that Excavators/Scrapers and Browsers are each confined to a single FG (1 and 5), while Grazers were dispersed across four (FGs 2, 5, 6, 8), highlighting high variability in behavioral and morphological traits (Figure 3, Table S5). Moreover, functional groups 1, 3, 4, 5, 6, and 7 were consistently present across all study plots, suggesting these species may be habitat generalists regarding benthic structure (Figure S4). Understanding the factors driving these functional patterns is crucial for comprehending the ecological roles of different species and informing targeted conservation efforts.

Principal Coordinate Analysis (PCoA) highlighted species with marked functional dissimilarity, occupying distinct regions in the multidimensional space (e.g., Spotted Moray *Gymnothorax meleagris* , Hawaii Whitespotted Toby *Canthigaster jactator* , and soldierfishes *Myripristis* spp.; Figure 4a). The loss of these species could therefore disproportionately reduce functional diversity and disrupt ecosystem dynamics (Hoey and Bellwood 2009; Mouillot et al. 2014), particularly because these species are predominant in FGs 9, 8, and 11, respectively. Supporting this, plots characterized by the lowest functional richness primarily lacked functional groups 9, 8, and 11 (Figure 4b). Although the functional roles of cryptic and nocturnal species remain poorly understood, prior studies suggest that a decline in fish functional richness and ecological complementarity can have significant and unexpected ramifications for ecosystem function (Bellwood et al., 2003). For example, the moray eel *G. meleagris* , a cryptic piscivore, plays a pivotal role in coral reef food webs both as predators and prey (Ineich et al. 2007).

The role of habitat complexity and live coral cover in shaping reef fish assemblages has been a central focus of research for decades (Luckhurst and Luckhurst 1978; Bell and Galzin 1984; Coker et al. 2014). Our analysis revealed that both 3D habitat complexity and coral morphology account for a significant amount of variability among a majority of the FGs observed in this study (Figure 5). The complexity metric of slope, in particular, exerted a strong positive influence on the abundance of six FGs, underscoring the importance of vertical habitat elevation for functionally diverse fish assemblages (Figure 5a). Higher slope values may indicate a greater availability of crevices and overhangs, which can offer refuge for prey species and shelter from strong currents (Fukunaga et al. 2020; Hench and Rosman 2013). Functional group 10, for example, which contains cryptic nocturnal species such as soldierfishes (Family Holocentridae) *Myripristis kuntee* and *Myripristis berndti* , is known to spend considerable time hiding beneath high ledges (Craig et al. 2007), supporting their strong association with higher slope values (Figure 5a). Functional groups 1 (Solitary Mobile) and 3 (Fusiform Predators) both exhibited significant negative responses to VRM, a metric of small‐scale complexity (figure 5a, Fukunaga and Burns 2020). FG1 was predominantly composed of larger invertivores and herbivores, such as wrasses (Family *Labridae*) and parrotfishes (Family *Scaridae*), whereas FG3 consisted of larger invertivores and piscivores, such as wrasses and trevally jacks (Family *Carangidae*; Figure 3 and Figure S5). This suggests that areas with higher VRM may pose challenges for these species in accessing algae or small prey, potentially impacting their foraging behavior (Rilov et al. 2007). Deciphering these nuances is essential for gaining a thorough understanding of reef ecology, which can, in turn, enhance the effectiveness of conservation practices in protecting key features that support ecosystem function.

While reef fish assemblages respond to both live coral cover and structural complexity (Pratchett et al. 2008; Komyakova et al. 2013), the specific roles of these two components in shaping fish community structure remain difficult to disentangle, as they are often tightly linked (Burns et al. 2019; Ferreira et al. 2023). To address these connections in our study, we used a post hoc Multivariate Redundancy Analysis (RDA) to examine how variation in coral morphology explained variation in 3D structural complexity metrics. While underlying reef structure also contributes to reef complexity, our results highlight that live coral morphology plays a distinct and measurable role in shaping structural habitat features, even in systems with moderate overall coral cover (Figure 7). Coral morphological composition significantly influenced structural complexity metrics (Figure 7), but these connections did not consistently align with fish assemblage patterns. Instead, the percent cover of specific coral morphologies showed stronger and broader associations with individual fish traits (Figure 6). For example, although mounding and branching corals contributed to surface complexity and VRM, respectively (Figure 7), their percent cover showed more numerous associations with individual fish traits than those structural metrics, with branching cover linked to 11 traits versus two for VRM (Figure 6). These findings highlight that the abundance of live coral potentially has a more substantial impact on fish assemblages than complexity metrics alone, supported by studies in the Great Barrier Reef (Komyakova et al. 2013). This underscores the importance of diverse coral morphologies that contribute to supporting functionally diverse reef ecosystems, with far‐reaching ramifications to the development of artificial reefs and coral restoration practices.

Reef fish have been shown to preferentially select certain corals for shelter based on morphology (Noonan et al. 2012; Kerry and Bellwood 2012; Kerry and Bellwood 2015; Wilson et al. 2016). Our study showed patterns of preference towards corals with a branching morphology, as the abundance of five FGs was significantly higher with increased cover of branching corals (Figure 5b). On the other hand, less‐complex corals like those with encrusting morphologies showed fewer and weaker associations (Figure 5b). The abundance of FG 5, composed of species with a Short/Deep body form and generally positioned in the Low water‐column, showed a strong positive relationship with branching corals (Figure 5b). This group consisted of surgeonfishes and damselfishes (Family Pomacentridae), including Hawaiian Dascyllus *Dascyllus albisella* , a highly territorial species that lives in hierarchical, conspecific groups and is known for its strong association with branching coral colonies (Noonan et al. 2012; Komyakova et al. 2013). Functional Group 10 (Nocturnal‐Cryptic) showed positive associations with knobby, branching, and encrusting corals, and a negative relationship with mounding corals, further highlighting the critical role that habitat structure plays for cryptic taxa (figure 5b, Depczynski and Bellwood 2004). Interestingly, FG 2 with sedentary cryptic species did not exhibit any significant relationships with either structural complexity or coral morphology. Non‐cryptic sedentary species (FG 8), however, did show a significant positive response to higher Surface Complexity values (Figure 5a). Observing cryptic species using conventional methods like UVC remains challenging in reef systems. Integrating environmental DNA (eDNA) could help overcome these limitations by improving detection of species presence and richness, which are often underestimated by visual surveys (Bessey et al. 2023). These results indicate that preserving a diverse array of morphological growth forms can help sustain reef functional biodiversity. Coral bleaching in Hawaiʻi disproportionately affects species such as *Pocillopora meandrina* and *Porites compressa* (McCutcheon and McKenna 2021; Winston et al. 2022), and understanding the loss of these key morphologies can help explain the affect on the functional diversity of reef fish communities.

Understanding species assemblages often requires analyzing multiple traits in combination (Winemiller and Rose 1992; Verberk et al. 2013). However, by assessing how habitat descriptors influence individual trait values, we isolated the contributions of each trait to functional group patterns and gained a more nuanced understanding of their interactions with 3D habitat features. For example, while trophic groups were not the primary determinants of functional groups, 6 out of the 8 trophic groups exhibited significant relationships with habitat characteristics (Figure 6), revealing patterns that were not apparent at the multi‐trait level. Piscivores, for instance, showed strong positive responses to higher surface complexity values (Figure 6). However, given that functional groups containing piscivores (FG1 and FG3) exhibited negative responses to complexity metrics such as VRM (Figure 5), this relationship is likely driven primarily by cryptic piscivores, such as eels, that actively exploit reef structures to access prey. In contrast, more mobile fusiform predators may be less suited to foraging in highly complex habitats. Browsers, on the other hand, increased with slope and branching corals but decreased with VRM and mounding corals (Figure 6). Piscivores and browsers play critical roles in regulating the food chain, cycling nutrients, and maintaining healthy algal coverage within the reef (Brandl et al. 2019). Understanding these dynamics is thus crucial for sustaining species that perform these essential ecological functions.

Schools and shoal formations are strategies for fish to reduce vulnerability to predation by increasing vigilance and decreasing the probability of mortality (Magurran 1990). As expected, schooling alone did not exhibit any significant associations with habitat metrics, suggesting that schooling may be their primary anti‐predator defense mechanism, as opposed to relying on refuges provided by the habitat's structural complexity (Eaton et al. 2016). The pairing trait (primarily exhibited in butterflyfishes) showed a negative relationship with branching corals, coinciding with studies highlighting the dependencies of these species on branching corals (Pratchett et al. 2008; Russ and Leahy 2017). Further, the abundance of solitary species was not associated with habitat metrics, which may be explained by solitary behavior being a trait exhibited by an array of species from cryptic and sedentary to larger mobile predators (Figure S4 and Table S5). While body size is often considered a ‘super‐trait’ across ecological disciplines due to its significant role in shaping animal‐habitat associations (Barneche et al. 2019; Glover et al. 2023), we did not find maximum body length to be statistically significant in driving fish functional groupings or explaining fish‐habitat relationships. However, recognizing the diverse ontogenetic dependencies exhibited by reef fish (Mellin et al. 2007), we acknowledge the constraints associated with relying solely on maximum body length rather than individual body length. These findings emphasize the intricacies of fish‐habitat interactions, and additional research can help elucidate the underlying mechanisms driving these patterns.

The ongoing degradation of coral reef habitats threatens the abundance and diversity of reef fish populations, with potential repercussions on ecosystem functions (Pratchett et al. 2014, 2018). Multiple studies have documented knock‐down effects on fish assemblages caused by various types of habitat degradation; however, the broader impact on ecosystem functions remains uncertain. To break down the mechanistic links between fish assemblages and ecological functioning, we used a comprehensive trait‐based approach that integrates trophic, behavioral, and morphological fish traits and evaluated how three‐dimensional habitat complexity and coral morphology influence fish functional assemblages. Notably, while 3D habitat complexity explained the abundance of more fish functional groups (Figure 5), coral morphology explained the abundance of twice as many individual fish traits (14 vs. 7; Figure 6). This finding suggests that live coral morphologies may have broader impacts on structuring reef fish functional assemblages. This highlights the distinct roles that live coral morphologies, including potential chemical signaling and nutrient exchange (Hay 2009; Holmes and Johnstone 2010), which are unlikely to be replicated by dead coral skeletons or artificial structures. As such, preserving a wide variety of live coral morphologies can help maintain functional reef ecosystems and can be used to inform conservation and restoration strategies.

The adoption of trait‐based approaches in ecology and conservation is gaining momentum, as these fields prioritize understanding ecosystem functioning and maintaining ecosystem services (Madin et al. 2016; Kissling et al. 2018; Barnett et al. 2019). Our study contributes to this effort by establishing a pathway linking fish assemblages with both coral and 3D habitat complexity using structure‐from‐motion photogrammetry techniques. Situated within a marine national park, our findings hold significant implications for conservation and monitoring efforts. We identified several plots, such as Plots 1, 5, 10, and 11, that stood out for their combination of high functional richness and elevated cover of complex coral morphologies (Figures 4b and Table S1). Notably, these sites are distributed throughout the park rather than clustered in a single spatial zone (Figure 1), suggesting that small‐scale structural features create dispersed functional hotspots across the reef system. Prioritizing protection of these locations can help preserve core areas of functional diversity. However, their broad spatial distribution also underscores the importance of conserving the entire park area, as adjacent and intermediate sites may enhance ecological connectivity and support processes such as dispersal, recruitment, and trophic interactions (McCook et al. 2009). This integrated approach enables informed prioritization while recognizing the interconnected nature of reef habitats and the fish communities they support.

## Author Contributions

**Sofia B. Ferreira:** conceptualization (equal), formal analysis (lead), investigation (lead), methodology (lead), writing – original draft (lead), writing – review and editing (equal). **John H. R. Burns:** conceptualization (equal), funding acquisition (equal), methodology (supporting), resources (lead), supervision (lead), writing – review and editing (equal). **Atsuko Fukunaga:** conceptualization (equal), methodology (supporting), writing – review and editing (equal). **Lillian J. Tuttle Raz:** methodology (supporting), writing – review and editing (equal). **Sheila A. McKenna:** investigation (supporting), writing – review and editing (equal). **Kailea Annandale:** writing – review and editing (equal). **Ryan J. Monello:** funding acquisition (equal), writing – review and editing (equal).

## Conflicts of Interest

The authors declare no conflicts of interest.

## Supporting information


**Data S1:** ece371992‐sup‐0001‐DataS1.docx.

## Data Availability

All data and statistical analysis supporting this study are publicly available through the National Park Service data repository and can be accessed at https://irma.nps.gov/DataStore/Reference/Profile/2308482. The R scripts used to compute the 3D habitat metrics are also available at https://github.com/AtsukoFukunaga/coral_reef_3Dmodels/tree/main.
