# Supplementary material for: 3D Habitat Complexity and Coral Morphology Modulate Reef Fish Functional Structure in a Marine National Park
Source: Ecol Evol. 2025 Sep 1;15(9):e71992. doi: 10.1002/ece3.71992 (PMC12401945; doi:10.1002/ece3.71992)
Supplement: Supplementary file 1 — Data S1: ece371992‐sup‐0001‐DataS1.docx. [file ECE3-15-e71992-s001.docx]

**Supplementary Materials**

**Fish Trait Categorization Methods**

**Body Form**

The body forms of fish species were derived from the "Morphology" section of FishBase, particularly using the ‘Body Shape Lateral’ description (Froese and Pauly, 2024). These shape categorizations are well-defined and have been described in detail by Beukhof et al. (2019), distinguishing between fusiform, elongated, flat, short and/or deep, and compressiform body types. In cases where body form information was unavailable for a specific species, genus-level descriptions were used as a proxy.

**Maximum Body Length**

Maximum body length was sourced from the 'Size' section of FishBase, which reports the maximum recorded total length (TL) of a species in centimeters.

**Trophic Group**

A species’ trophic group reflects both its diet and feeding behavior. We categorized species into the following groups: Omnivore (feeds on both animals and algae), Piscivore (feeds on fish), Invertivore (feeds on invertebrates), Planktivore (feeds on plankton), Corallivore (feeds on coral), Browser (feeds on macroalgae), Excavator/Scraper (feeds on epilithic algal turf), and Grazer/Detritivore (feeds on algal turf and detritus). These classifications were systematically assigned based on detailed information found in the Biology and Trophic Ecology sections of FishBase, particularly in the section detailing food items consumed by each species (Froese and Pauly, 2024).

**Water Column Position**

We categorized species by their water column position using three categories: Bottom (typically <1 meter above the substrate), Low (generally between 1 and 4 meters from the substrate), and High (more than 4 meters above the substrate). These categories were derived through a comprehensive review of species-specific information from the FishBase database, supplemented by relevant ecological and biological literature. Where species-specific data were unavailable in FishBase, we relied on genus-level characterizations to maintain methodological consistency. For species classified as "benthopelagic" in FishBase—indicative of species that inhabit both benthic (near-bottom) and midwater zones (Froese and Pauly, 2024)—we assigned them to the category that best reflected their predominant behavior in natural habitats, as supported by the literature and field observations. For instance, *Plectroglyphidodon johnstonianus* is categorized as benthopelagic in FishBase, yet literature indicates that this species exhibits a strong affinity for remaining close to shelter within the reef (Randall, 2010; Hobson, 1972). Therefore, it was categorized under the "Bottom" group. Similarly, species explicitly described as "benthic" in FishBase were placed in the "Bottom" category. For example, *Paracirrhites arcatus* is listed in FishBase as a benthic species (Froese and Pauly, 2024; Mundy, 2005). On the other hand, species exhibiting a higher position in the water column, such as *Melichthys niger*, were categorized as "High." FishBase describes this species as "swimming high above the substrate while feeding on zooplankton" (Kuiter & Tonozuka, 2001), reflecting its typical habitat use and supporting its placement in the "High" category.

**Relative Mobility**

We categorized fish species according to their daily mobility patterns into three categories: Sedentary, Mobile, and Wide. Since FishBase does not have a specific classification for daily mobility or home range, we inferred mobility based on species-specific behaviors described in the Biology or Ecology sections of FishBase, supplemented by relevant literature, field observations, and ecological knowledge.

Sedentary species typically exhibit very limited daily movement, often confining themselves to a small, well-defined home range or territory on the reef. For example, *Plectroglyphidodon imparipennis* is described in FishBase as staying "close to the shelter of small holes or sea urchin furrows in bare rock" (Myers, 1991). Similarly, *Paracirrhites forsteri* is noted to "perch on the outermost branches of *Stylophora*, *Pocillopora*, and *Acropora* corals" (Lieske & Myers, 1994), and *Plagiotremus ewaensis* is noted to “hover above the reef waiting for unwary prey” (Mundy, 2005). Based on these behaviors, these species were categorized as Sedentary.

Mobile species regularly swim within the reef but tend to remain within a limited range, without traveling between different reef systems. These species are often generalist foragers that traverse the reef to access diverse feeding opportunities while staying restricted to one reef system. For example, *Scarus rubroviolaceus* is described in FishBase as a "roving herbivore" (Sommer et al., 1996; Bellwood and Choat, 1990), meaning it forages across reef patches but does not migrate large distances daily, and was therefore categorized as Mobile.

Wide species exhibit more extensive daily movements, often covering long distances across multiple reef systems or open water. These species tend to be larger, more active swimmers capable of significant locomotion for foraging, reproduction, or migration. For example, *Caranx melampygus* (trevally) is listed in FishBase as inhabiting "pelagic coastal and oceanic waters," indicating it swims across large distances spanning both reef and open water habitats, warranting its classification as Wide (Mundy, 2005).

**Crypsis**

Species exhibiting either behavioral or morphological crypsis (camouflage) were categorized as cryptic. Behavioral crypsis refers to species that modify their behavior to avoid detection, such as hiding in crevices or remaining motionless during the day, while morphological crypsis involves coloration or patterns that blend into the environment. For nocturnal species that employ diurnal inactivity and cryptic behavior (*Myripristis spp.*), we chose to categorize them as cryptic. This allowed us to maintain consistency in characterizing daytime traits across all species in the study, regardless of their primary activity period.

**Activity Period**

Activity period characterizes whether a species is most active during the day (diurnal) or at night (nocturnal). These classifications were primarily made based on information in the Biology and Ecology sections of FishBase. In cases where no remarks regarding the activity period were available, we utilized additional literature to inform our classifications.

**Coral Morphology Categorization Methods**

The classification of each colony follows the definitions and naming conventions outlined in Winston et al. (2020). To demonstrate how these classifications were applied in our study, the following images provide visual examples for each primary morphology from our survey plots.

**Branching**: Form distinct tree-like branches that extend upward from a central base.


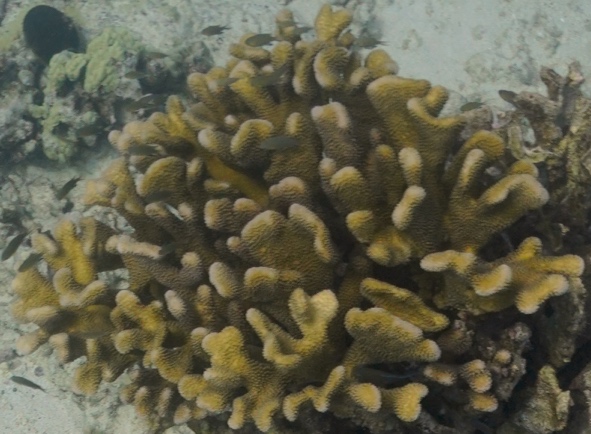


**Mounding**: Form solid, spherical, or large ellipsoid (ball-shaped) colonies.


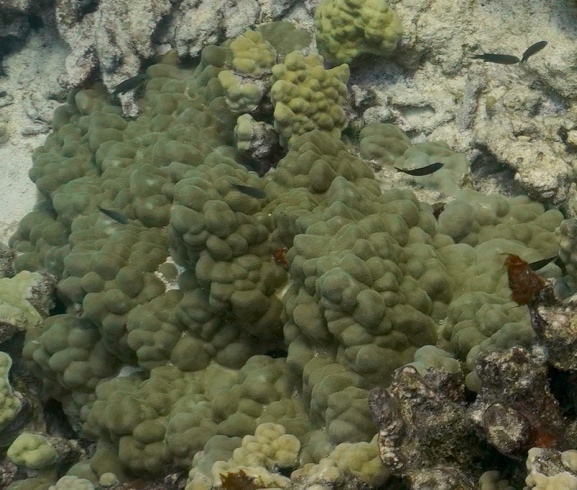


**Knobby**: Form short, thick, stubby branches, which often resemble knuckles.


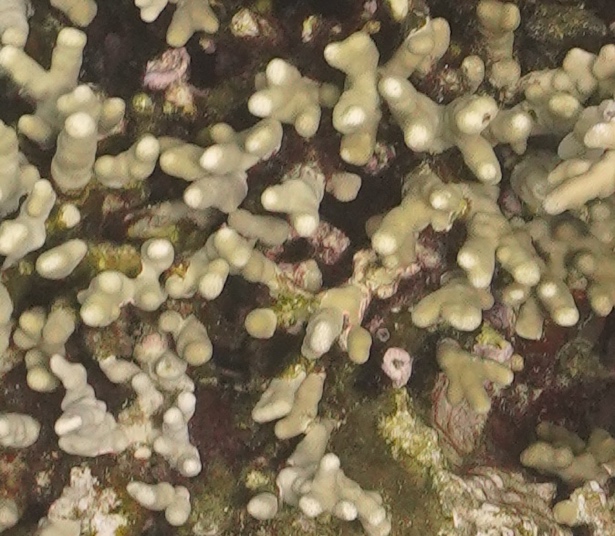


**Encrusting**: Form a thin layer that adheres closely to the benthic substrate.


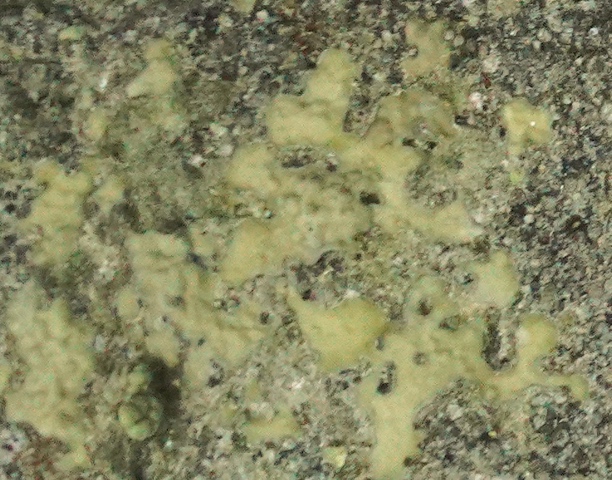


**Table S1.** Percent cover of each live coral morphology type, mean three-dimensional colony area, and total live coral percent cover for each survey plot.

| Site ID | Branching (%) | Encrusting (%) | Mounding (%) | Knobby (%) | 3D Area (m²) | Total Live Coral (%) |
| --- | --- | --- | --- | --- | --- | --- |
| 1 | 0.0007 | 2.46 | 2.60 | 0.004 | 0.002 | 5.08 |
| 2 | 0.28 | 0 | 1.38 | 0.366 | 0.008 | 2.04 |
| 3 | 2.56 | 2.95 | 13.2 | 0.36 | 0.006 | 19.3 |
| 4 | 1.54 | 0.45 | 11.9 | 1.14 | 0.01 | 15.0 |
| 5 | 6.19 | 0.99 | 18.45 | 2.97 | 0.02 | 28.6 |
| 6 | 0.04 | 0.002 | 5.49 | 0.08 | 0.009 | 5.63 |
| 7 | 0 | 1.56 | 3.77 | 0.61 | 0.005 | 5.95 |
| 8 | 0.01 | 6.56 | 0.53 | 0.005 | 0.002 | 7.11 |
| 9 | 0.04 | 1.83 | 0.25 | 0 | 0.001 | 2.13 |
| 10 | 6.03 | 1.69 | 8.67 | 6.63 | 0.02 | 23.0 |
| 11 | 4.12 | 1.99 | 12.9 | 7.3 | 0.02 | 26.4 |
| 12 | 8.23 | 0 | 10.9 | 0 | 0.01 | 19.2 |
| 13 | 0.007 | 0.01 | 2.06 | 0.002 | 0.004 | 2.08 |
| 14 | 0.003 | 1.13 | 0.68 | 0.0005 | 0.001 | 1.82 |
| 15 | 0.04 | 6.17 | 0.61 | 0.004 | 0.005 | 6.83 |
| 16 | 0.24 | 0.70 | 3.90 | 0.37 | 0.01 | 5.22 |
| 17 | 0.06 | 6.19 | 5.01 | 0.05 | 0.004 | 11.3 |
| 18 | 0.03 | 9.78 | 1.71 | 0.005 | 0.002 | 11.5 |
| 19 | 0.01 | 1.12 | 5.09 | 0 | 0.004 | 6.22 |


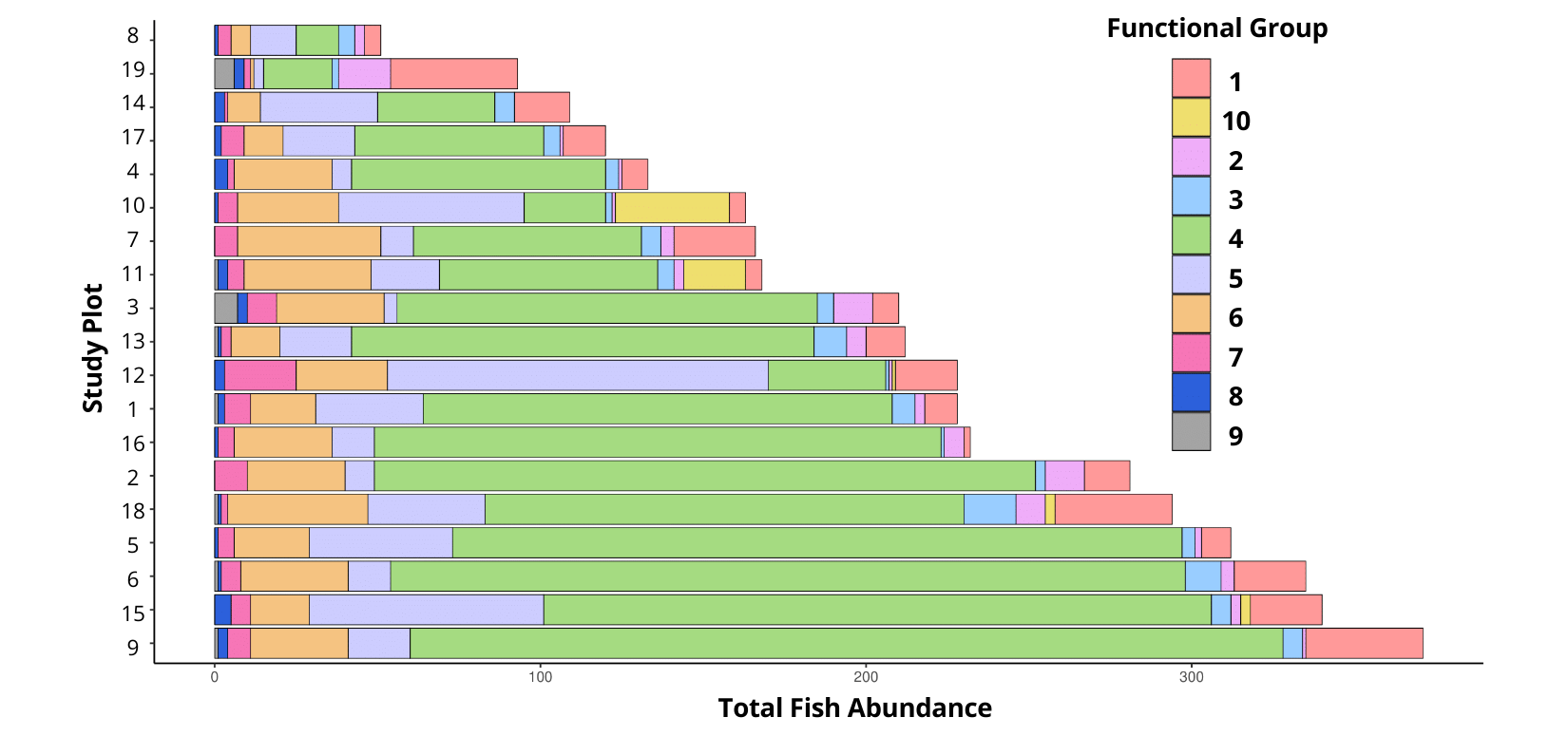


**Figure S1.** Stacked bar charts showing total fish abundance by plot, colored by the abundance proportion of each cluster.


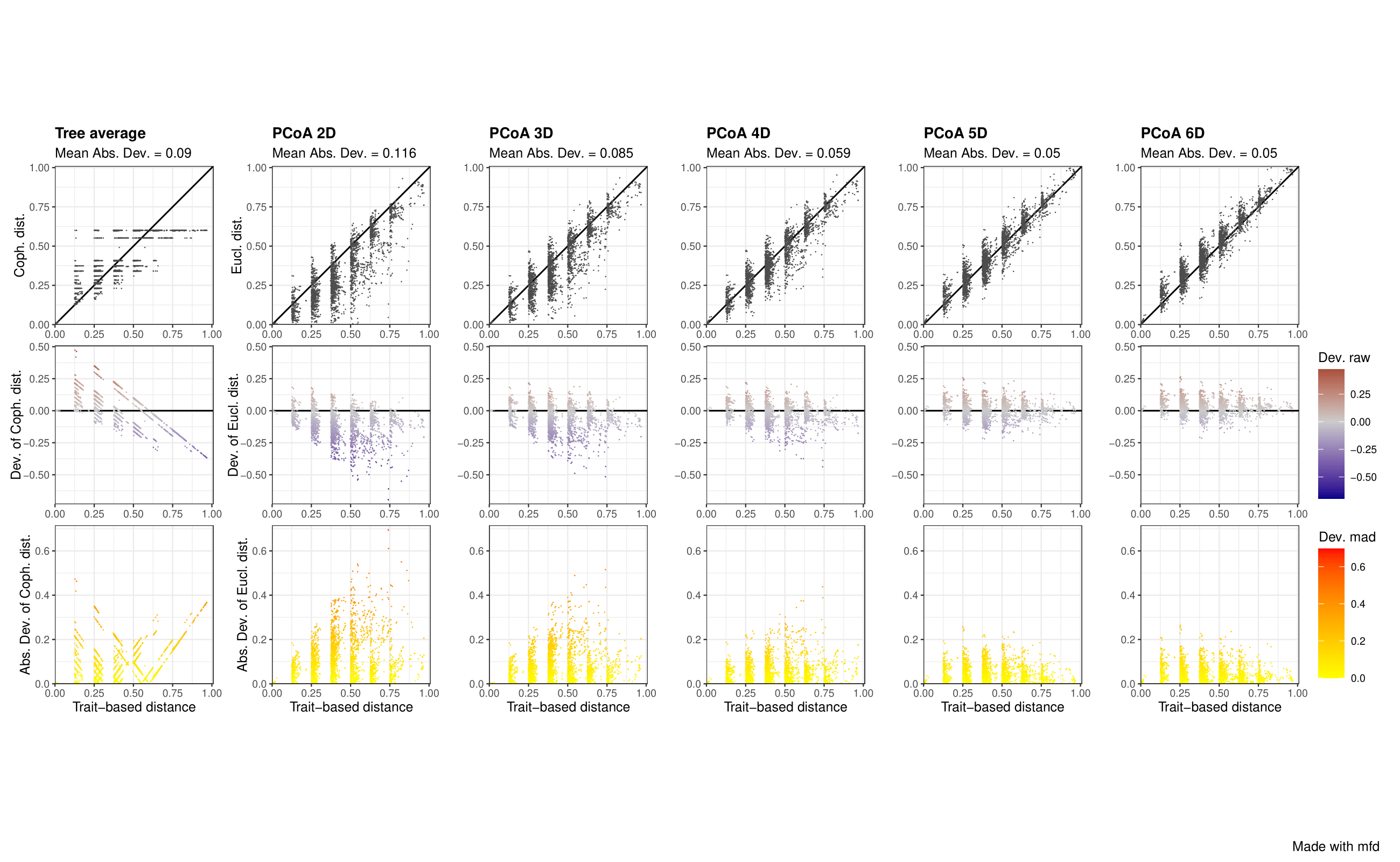


**Figure S2.** Scatterplots showing the quality of the multidimensional trait space with increasing number of PCoA axes. The first row depicts species functional distances in the multidimensional space. The second row shows the raw deviation of species distances in the functional space compared to trait-based distances. The third row shows the absolute deviation of the distance in the functional space. The mean absolute deviations (MADs) between the original trait-based distances and the Euclidean distances in the functional space are indicated at the top for each number of PCoA dimensions, with lowest values indicating a more robust functional space.


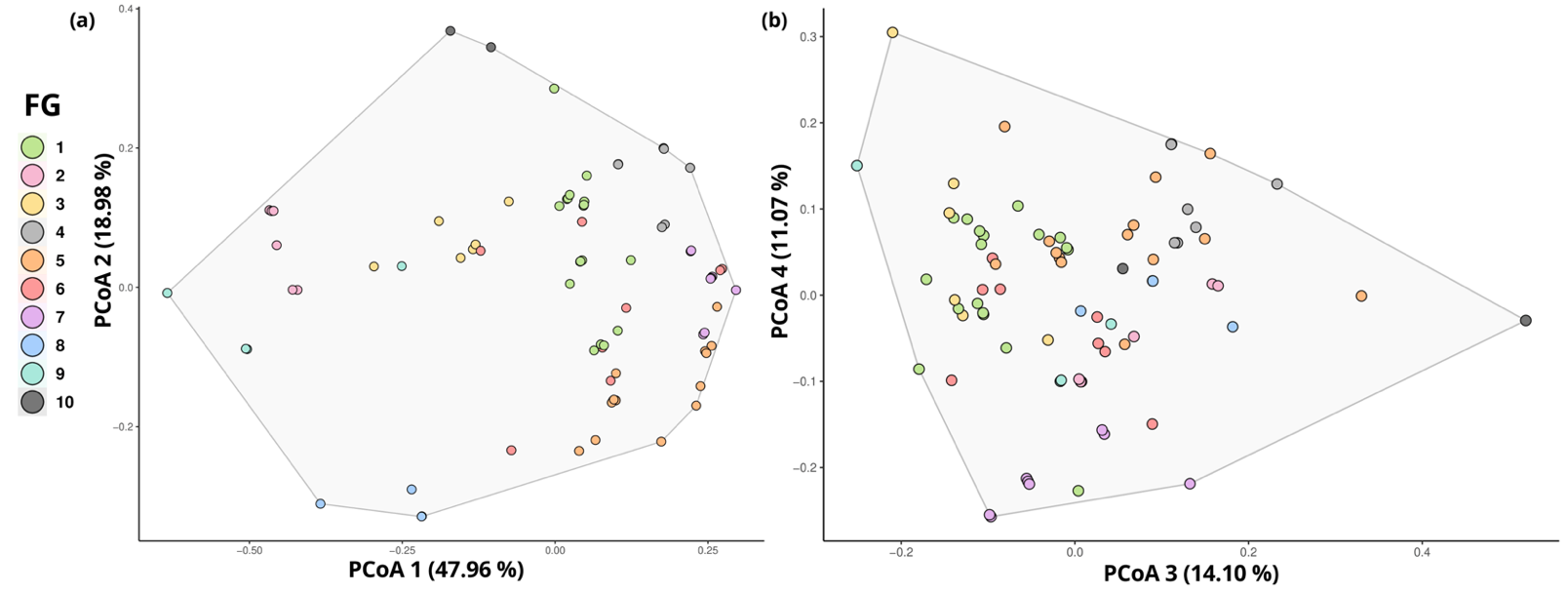
**Figure S3.** PCoA biplots showing the fish functional space with (a) axis 1 explaining 47.96% and axis 2 explaining 18.98%, and (b) axis 3 explaining an additional 14.10% and axis 4 explaining 11.07%. Points are colored by functional groups obtained from hierarchical clustering.

**
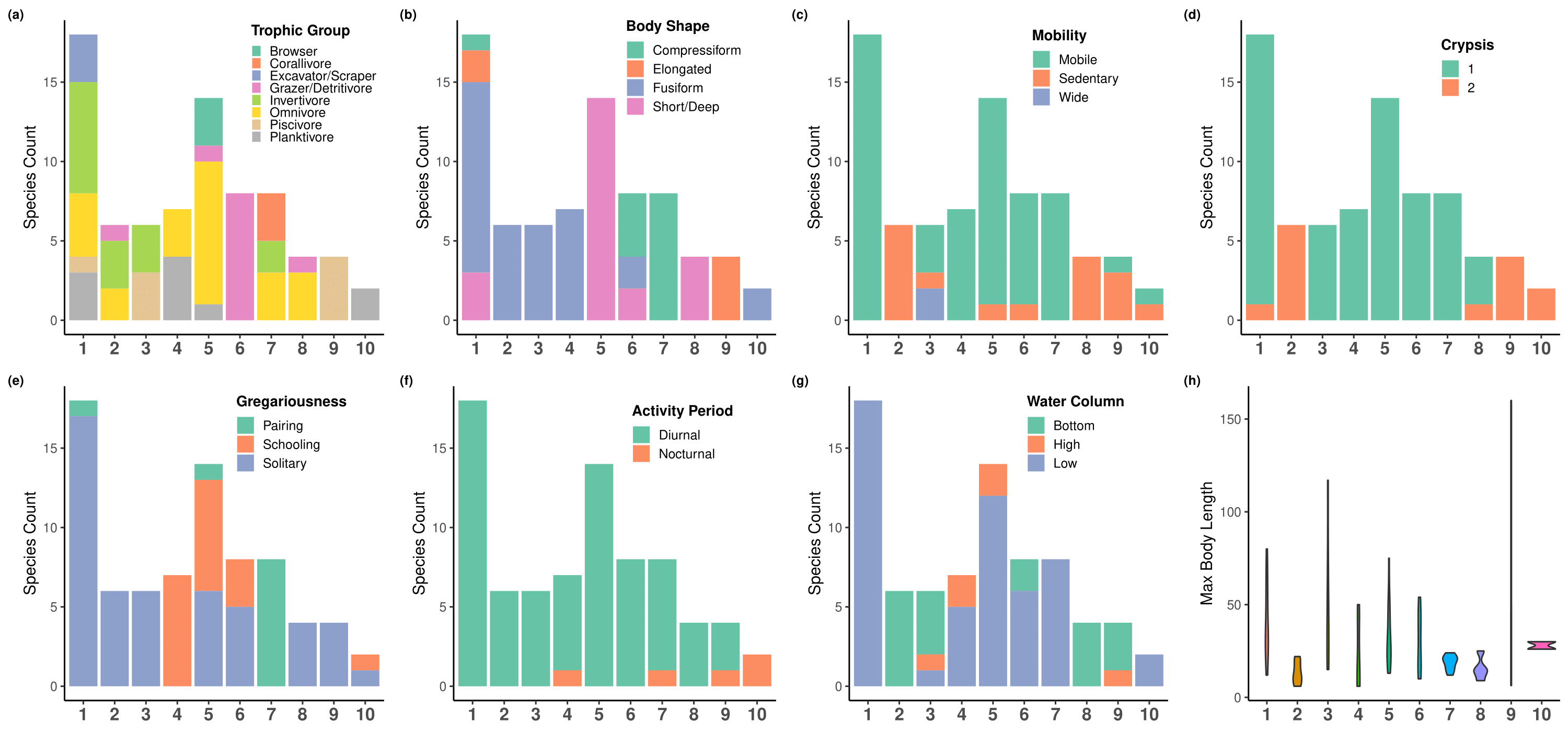
Figure S4.** Stacked bar charts showing the number of species associated with each trait value across clusters for (a) Trophic Group, (b) Body Shape, (c) Mobility, (d) Crypsis, (e) Gregariousness, (f) Activity Period, and (g) Water Column. (h) Violin plot showing distribution of Max Body Length among clusters

**Supplementary Table S2.** Strength of the correlations between traits and PCoA axes. For ordinal and nominal traits, the value of the Kruskal-Wallis test indicates relative importance. For continuous traits, the goodness-of-fit (r^2^) of a linear regression model is indicated. Significance levels (p) are reported.

| Axis | Trait | Test | Statistic | P-value |
| --- | --- | --- | --- | --- |
| PCoA 1 | Gregariousness | Kruskal-Wallis (n^2^) | 0.527 | <0.0001 |
|  | Water Column | Kruskal-Wallis (n^2^) | 0.487 | <0.0001 |
|  | Body Shape | Kruskal-Wallis (n^2^) | 0.421 | <0.0001 |
|  | Mobility | Kruskal-Wallis (n^2^) | 0.477 | <0.0001 |
|  | Crypsis | Kruskal-Wallis (n^2^) | 0.387 | <0.0001 |
|  | Trophic Group | Kruskal-Wallis (n^2^) | 0.295 | <0.001 |
| PCoA 2 | Body Shape | Kruskal-Wallis (n^2^) | 0.786 | <0.0001 |
|  | Trophic Group | Kruskal-Wallis (n^2^) | 0.391 | <0.0001 |
| PCoA 3 | Gregariousness | Kruskal-Wallis (n^2^) | 0.393 | <0.0001 |
|  | Trophic Group | Kruskal-Wallis (n^2^) | 0.387 | <0.001 |
|  | Activity Period | Kruskal-Wallis (n^2^) | 0.083 | <0.001 |
|  | Mobility | Kruskal-Wallis (n^2^) | 0.171 | <0.01 |
|  | Max Length | Linear Model (r^2^) | 0.128 | <0.05 |
|  | Crypsis | Kruskal-Wallis (n^2^) | 0.048 | <0.05 |
| PCoA 4 | Water Column | Kruskal-Wallis (n^2^) | 0.244 | <0.001 |
|  | Trophic Group | Kruskal-Wallis (n^2^) | 0.342 | <0.01 |
|  | Body Shape | Kruskal-Wallis (n^2^) | 0.360 | <0.001 |
|  | Max Length | Linear Model (r^2^) | 0.195 | <0.001 |
|  | Crypsis | Kruskal-Wallis (n^2^) | 0.067 | <0.05 |
|  | Mobility | Kruskal-Wallis (n^2^) | 0.129 | <0.001 |
|  | Gregariousness | Kruskal-Wallis (n^2^) | 0.342 | <0.0001 |

**Table S3.** Coefficient estimates for negative binomial generalized linear models (GLM). Columns depict response variables (Functional Group Abundance), and rows represent predictor variables selected through AIC model selection. Asterisks indicate significance level (* = p<0.05, ** = p<0.01, *** = p<0.001). Pseudo-R^2^ was calculated as 1-(residual deviance/null deviance).

| **Habitat Feature** | **Predictor** | **FG 1** | **FG 3** | **FG 5** | **FG 6** | **FG 7** | **FG 8** | **FG 9** | **FG 10** |
| --- | --- | --- | --- | --- | --- | --- | --- | --- | --- |
| **Structural Complexity** | Curvature | 0.10 |  |  |  |  | 0.06 | 0.01* |  |
|  | Surface Complexity |  |  |  |  |  | 0.02* | 0.02* |  |
|  | Slope |  | 0.01* | 0.001** | 0.0007*** |  | 0.02* | 0.03* | 0.006** |
|  | VRM | 0.00371 ** | 0.0005*** | 0.05 |  | 0.003** |  |  |  |
|  | Intercept | < 2e^-16^ *** | < 2e^-16^ *** | < 2e^-16^ *** | < 2e^-16^ *** | < 2e^-16^ *** | 0.0004*** | 0.42 | 0.59 |
|  | Pseudo-R^2^ | 0.32 | 0.38 | 0.36 | 0.29 | 0.31 | 0.28 | 0.21 | 0.45 |
|  | Log-Likelihood | -131.771 | -88.3 | -156.2 | -145.3 | -94.92 | -57.9 | -46.9 | -49.28 |
| **Coral** | Encrusting |  | 0.09 | 0.03* |  |  |  |  | 0.002** |
|  | Mounding |  |  | 0.0002*** |  |  |  | 0.01* | 0.03* |
|  | Branching |  | 0.03* | 4.06e^-08^ *** |  | 5.31e^-05^ *** |  | 0.03* | 0.005** |
|  | Knobby | 0.005** |  |  |  |  |  |  | 1.2e^-07^** |
|  | 3D Area |  |  |  |  | 0.01* |  |  |  |
|  | Intercept | < 2e^-16^ *** | < 2e^-16^ *** | < 2e^-16^ *** |  | < 2e^-16^ *** |  | 0.16 | 0.02* |
|  | Pseudo-R^2^ | 0.27 | 0.36 | 0.64 |  | 0.48 |  | 0.35 | 0.9 |
|  | Log-Likelihood | -132.9 | -88.6 | -145.3 |  | -90.2 |  | -43.98 | -28.4 |

**Table S4.** Coefficient estimates for negative binomial generalized linear models (GLM). Columns depict response variables (Functional Group Abundance), and rows represent predictor variables selected through AIC model selection. Asterisks indicate significance level (* = p<0.05, ** = p<0.01, *** = p<0.001). Abbreviations: Brows=Browser, Pisci=Piscivore, Omni=Omnivore, Gr/Det=Grazer/Detritivore, Exc/Scr=Excavator/Scraper, Inver=Invertivore, Sedent=Sedentary, Noct=Nocturnal, Sh/De=Short/Deep, Comp=Compressiform, Cryp=Cryptic, Elon=Elongated, Corall=Corallivore.

| **Habitat Feature** | **Predictor** | **Brows** | **Pisci** | **Omni** | **Gr/Det** | **Exc/Scr** | **Inver** | **Bottom** | **Low** | **Sedent** | **Noct** | **Pair** | **Sh/De** | **Comp** | **Cryp** | **Elon** | **Corall** |
| --- | --- | --- | --- | --- | --- | --- | --- | --- | --- | --- | --- | --- | --- | --- | --- | --- | --- |
| Structural Complexity | Curvature |  | 0.1 |  |  | 0.09 |  |  |  |  |  |  |  |  |  |  |  |
|  | Surface Complexity |  | 0.02* |  |  | 0.02* |  |  |  |  |  |  |  |  |  |  |  |
|  | Slope | 0.001** | 0.03* |  | 0.003** | 0.04* | 0.03* |  | 0.007** |  | 0.005** |  | 0.0002*** |  |  |  |  |
|  | VRM | 0.05 |  |  |  |  | 0.02* |  |  |  | 0.16 |  |  |  |  |  |  |
|  | Intercept | < 2e-^16^ *** | < 2e-^16^ *** |  | < 2e^-16^ *** | 4.37e^-06^ *** | < 2e^-16^ *** |  | < 2e^-16^ *** |  | 0.01* |  | < 2e^-16^ *** |  |  |  |  |
|  | Pseudo-R^2^ | 0.40 | 0.23 |  | 0.26 | 0.22 | 0.24 |  | 0.29 |  | 0.46 |  | 0.40 |  |  |  |  |
|  | Log- Likelihood | -137.7 | -77.0 |  | -143.1 | -87.5 | -131.5 |  | -177.8 |  | -84.7 |  | -165.89 |  |  |  |  |
| Coral | Mounding | 0.001** |  |  |  | 0.01* |  | 0.0009*** | 0.0008*** |  | 0.07 |  | 0.004** |  |  | 0.01* |  |
|  | Branching | 1.74e^-07^ *** |  | 0.003** |  | 0.04* | 0.02* | 0.0008*** | 2.1e^-08^ *** |  | 0.0001  *** | 1.65e^-06^ *** | 4.14e^-07^ *** |  |  | 0.15 | 0.02* |
|  | Encrusting | 0.03* |  | 0.01* |  | 0.06 |  |  | 0.02* |  | 0.05 |  | 0.11 |  |  |  |  |
|  | Knobby |  |  | 5.06e^-05^ *** |  | 0.11 |  | 0.01* |  | 0.03* | 0.0004*** | 0.001** |  | 0.002** | 0.04* |  |  |
|  | 3D Area |  |  |  |  |  |  |  |  |  | 0.04* |  |  |  |  |  |  |
|  | Intercept | < 2e-16 *** |  | < 2e^-16^ *** |  | 0.0001  *** | < 2e^-16^ *** | < 2e^-16^ *** | < 2e^-16^ *** | < 2e^-16^ *** | 0.02* | < 2e^-16^ *** | < 2e^-16^ *** | < 2e^-16^ *** | < 2e^-16^ *** | 0.15 | < 2e^-16^ *** |
|  | Pseudo-R^2^ | 0.60 |  | 0.58 |  | 0.43 | 0.18 | 0.58 | 0.65 | 0.23 | 0.69 | 0.61 | 0.61 | 0.28 | 0.21 | 0.27 | 0.25 |
|  | Log- Likelihood | -130 |  | -142.3 |  | -81.4 | -132.9 | -130.5 | -163.9 | -129.0 | -76.5 | -85.4 | -157.6 | -138.3 | -124.5 | -63.1 | -68.7 |

| **Table S5**. List of all fish species recorded with associated functional traits and cluster grouping. | | | | | | | | |  |
| --- | --- | --- | --- | --- | --- | --- | --- | --- | --- |
| Species | Max Length | Trophic Group | Crypsis | Mobility | Gregariousness | Water Column | Body Shape | Activity Period | cluster |
| Abudefduf abdominalis | 30 | Omnivore | 1 | Mobile | Schooling | Low | Short/Deep | Diurnal | 5 |
| Abudefduf vaigiensis | 20 | Omnivore | 1 | Mobile | Schooling | High | Short/Deep | Diurnal | 5 |
| Acanthurus blochii | 45 | Grazer/Detritivore | 1 | Mobile | Schooling | Low | Compressiform | Diurnal | 6 |
| Acanthurus dussumieri | 54 | Grazer/Detritivore | 1 | Mobile | Schooling | Low | Compressiform | Diurnal | 6 |
| Acanthurus nigrofuscus | 21 | Grazer/Detritivore | 1 | Mobile | Schooling | Bottom | Compressiform | Diurnal | 6 |
| Acanthurus olivaceus | 35 | Grazer/Detritivore | 1 | Mobile | Solitary | Low | Compressiform | Diurnal | 6 |
| Acanthurus thompsoni | 27 | Planktivore | 1 | Mobile | Solitary | Low | Compressiform | Diurnal | 1 |
| Aphareus furca | 70 | Piscivore | 1 | Wide | Solitary | Low | Fusiform | Diurnal | 3 |
| Aulostomus chinensis | 80 | Omnivore | 1 | Mobile | Solitary | Low | Elongated | Diurnal | 1 |
| Balistes polylepis | 76 | Invertivore | 1 | Mobile | Solitary | Low | Short/Deep | Diurnal | 1 |
| Bodianus albotaeniatus | 32.8 | Invertivore | 1 | Mobile | Solitary | Bottom | Fusiform | Diurnal | 3 |
| Calotomus carolinus | 54 | Excavator/Scraper | 1 | Mobile | Solitary | Low | Fusiform | Diurnal | 1 |
| Cantherhines dumerilii | 38 | Invertivore | 1 | Mobile | Solitary | Low | Short/Deep | Diurnal | 1 |
| Canthigaster jactator | 9 | Omnivore | 2 | Sedentary | Solitary | Bottom | Short/Deep | Diurnal | 8 |
| Caranx melampygus | 117 | Piscivore | 1 | Wide | Solitary | High | Fusiform | Diurnal | 3 |
| Centropyge loricula | 15 | Omnivore | 1 | Sedentary | Solitary | Bottom | Short/Deep | Diurnal | 8 |
| Centropyge potteri | 10 | Grazer/Detritivore | 1 | Mobile | Solitary | Bottom | Short/Deep | Diurnal | 6 |
| Cephalopholis argus | 60 | Piscivore | 1 | Mobile | Solitary | Low | Fusiform | Diurnal | 1 |
| Chaetodon auriga | 21 | Omnivore | 1 | Mobile | Pairing | Low | Compressiform | Diurnal | 7 |
| Chaetodon kleinii | 15 | Omnivore | 1 | Mobile | Pairing | Low | Compressiform | Diurnal | 7 |
| Chaetodon lunula | 20 | Omnivore | 1 | Mobile | Pairing | Low | Compressiform | Nocturnal | 7 |
| Chaetodon multicinctus | 12 | Corallivore | 1 | Mobile | Pairing | Low | Compressiform | Diurnal | 7 |
| Chaetodon ornatissimus | 20 | Corallivore | 1 | Mobile | Pairing | Low | Compressiform | Diurnal | 7 |
| Chaetodon quadrimaculatus | 16 | Corallivore | 1 | Mobile | Pairing | Low | Compressiform | Diurnal | 7 |
| Chlorurus spilurus | 48.6 | Excavator/Scraper | 1 | Mobile | Solitary | Low | Fusiform | Diurnal | 1 |
| Chromis hanui | 6 | Planktivore | 1 | Mobile | Schooling | Low | Fusiform | Diurnal | 4 |
| Chromis ovalis | 15 | Planktivore | 1 | Mobile | Schooling | Low | Fusiform | Diurnal | 4 |
| Chromis pacifica | 7.6 | Planktivore | 1 | Mobile | Schooling | High | Fusiform | Diurnal | 4 |
| Chromis vanderbilti | 6 | Planktivore | 1 | Mobile | Schooling | High | Fusiform | Diurnal | 4 |
| Cirripectes vanderbilti | 10 | Grazer/Detritivore | 2 | Sedentary | Solitary | Bottom | Fusiform | Diurnal | 2 |
| Coris gaimard | 40 | Omnivore | 1 | Mobile | Solitary | Low | Fusiform | Diurnal | 1 |
| Coris venusta | 19.3 | Invertivore | 1 | Mobile | Solitary | Low | Fusiform | Diurnal | 1 |
| Ctenochaetus hawaiiensis | 25 | Browser | 1 | Mobile | Solitary | Low | Short/Deep | Diurnal | 5 |
| Ctenochaetus strigosus | 15 | Grazer/Detritivore | 1 | Mobile | Solitary | Low | Short/Deep | Diurnal | 6 |
| Dascyllus albisella | 13 | Omnivore | 1 | Sedentary | Schooling | Low | Short/Deep | Diurnal | 5 |
| Fistularia commersonii | 160 | Piscivore | 2 | Mobile | Solitary | High | Elongated | Diurnal | 9 |
| Forcipiger flavissimus | 22 | Invertivore | 1 | Mobile | Pairing | Low | Compressiform | Diurnal | 7 |
| Forcipiger longirostris | 24 | Invertivore | 1 | Mobile | Pairing | Low | Compressiform | Diurnal | 7 |
| Gomphosus varius | 30 | Omnivore | 1 | Mobile | Solitary | Low | Fusiform | Diurnal | 1 |
| Gymnothorax meleagris | 120 | Piscivore | 2 | Sedentary | Solitary | Bottom | Elongated | Nocturnal | 9 |
| Halichoeres ornatissimus | 18 | Invertivore | 1 | Sedentary | Solitary | Bottom | Fusiform | Diurnal | 3 |
| Kyphosus sp | 53 | Grazer/Detritivore | 1 | Mobile | Schooling | Low | Short/Deep | Diurnal | 5 |
| Labroides phthirophagus | 12 | Invertivore | 1 | Mobile | Solitary | Low | Elongated | Diurnal | 1 |
| Lutjanus kasmira | 49 | Omnivore | 1 | Mobile | Schooling | Low | Fusiform | Nocturnal | 4 |
| Macropharyngodon geoffroy | 15 | Invertivore | 1 | Mobile | Solitary | Low | Fusiform | Diurnal | 1 |
| Melichthys niger | 50 | Omnivore | 1 | Mobile | Solitary | High | Short/Deep | Diurnal | 5 |
| Melichthys vidua | 40 | Omnivore | 1 | Mobile | Solitary | Low | Short/Deep | Diurnal | 5 |
| Monotaxis grandoculis | 60 | Planktivore | 1 | Mobile | Solitary | Low | Fusiform | Diurnal | 1 |
| Myripristis berndti | 30 | Planktivore | 2 | Mobile | Solitary | Low | Fusiform | Nocturnal | 10 |
| Myripristis kuntee | 26 | Planktivore | 2 | Sedentary | Schooling | Low | Fusiform | Nocturnal | 10 |
| Naso hexacanthus | 75 | Planktivore | 1 | Mobile | Schooling | Low | Short/Deep | Diurnal | 5 |
| Naso lituratus | 46 | Browser | 1 | Mobile | Schooling | Low | Short/Deep | Diurnal | 5 |
| Novaculichthys taeniourus | 30 | Invertivore | 2 | Mobile | Pairing | Low | Fusiform | Diurnal | 1 |
| Ostracion meleagris | 25 | Grazer/Detritivore | 1 | Sedentary | Solitary | Bottom | Short/Deep | Diurnal | 8 |
| Oxycheilinus unifasciatus | 46 | Piscivore | 1 | Mobile | Solitary | Bottom | Fusiform | Diurnal | 3 |
| Paracirrhites arcatus | 20 | Invertivore | 2 | Sedentary | Solitary | Bottom | Fusiform | Diurnal | 2 |
| Paracirrhites forsteri | 22 | Omnivore | 2 | Sedentary | Solitary | Bottom | Fusiform | Diurnal | 2 |
| Parupeneus cyclostomus | 50 | Omnivore | 1 | Mobile | Schooling | Low | Fusiform | Diurnal | 4 |
| Parupeneus insularis | 30.3 | Invertivore | 1 | Mobile | Solitary | Low | Fusiform | Diurnal | 1 |
| Parupeneus multifasciatus | 35 | Omnivore | 1 | Mobile | Schooling | Low | Fusiform | Diurnal | 4 |
| Pervagor spilosoma | 18 | Omnivore | 1 | Mobile | Solitary | Low | Short/Deep | Diurnal | 5 |
| Plagiotremus ewaensis | 10.2 | Piscivore | 2 | Sedentary | Solitary | Bottom | Elongated | Diurnal | 9 |
| Plagiotremus goslinei | 6.4 | Piscivore | 2 | Sedentary | Solitary | Bottom | Elongated | Diurnal | 9 |
| Plectroglyphidodon imparipennis | 6 | Omnivore | 2 | Sedentary | Solitary | Bottom | Fusiform | Diurnal | 2 |
| Plectroglyphidodon johnstonianus | 14 | Omnivore | 1 | Sedentary | Solitary | Bottom | Short/Deep | Diurnal | 8 |
| Pseudocheilinus evanidus | 9 | Invertivore | 2 | Sedentary | Solitary | Bottom | Fusiform | Diurnal | 2 |
| Pseudocheilinus octotaenia | 14 | Invertivore | 2 | Sedentary | Solitary | Bottom | Fusiform | Diurnal | 2 |
| Scarus psittacus | 43 | Grazer/Detritivore | 1 | Mobile | Solitary | Low | Fusiform | Diurnal | 6 |
| Scarus rubroviolaceus | 70 | Excavator/Scraper | 1 | Mobile | Solitary | Low | Fusiform | Diurnal | 1 |
| Stegastes fasciolatus | 16.5 | Grazer/Detritivore | 1 | Sedentary | Solitary | Low | Fusiform | Diurnal | 6 |
| Stethojulis balteata | 15 | Invertivore | 1 | Mobile | Solitary | Bottom | Fusiform | Diurnal | 3 |
| Sufflamen bursa | 25 | Omnivore | 1 | Mobile | Solitary | Low | Short/Deep | Diurnal | 5 |
| Thalassoma duperrey | 28 | Omnivore | 1 | Mobile | Solitary | Low | Fusiform | Diurnal | 1 |
| Xanthichthys auromarginatus | 30 | Planktivore | 1 | Mobile | Solitary | Low | Short/Deep | Diurnal | 1 |
| Zanclus cornutus | 23 | Omnivore | 1 | Mobile | Pairing | Low | Short/Deep | Diurnal | 5 |
| Zebrasoma flavescens | 20 | Browser | 1 | Mobile | Schooling | Low | Short/Deep | Diurnal | 5 |

**References**

Bellwood, D. R., & Choat, J. H. (1990). A functional analysis of grazing in parrotfishes (family Scaridae): The ecological implications. Environ. Biol. Fishes.

Beukhof, E., Dencker, T. S., Palomares, M. L. D., & Maureaud, A. (2019). A trait collection of marine fish species from North Atlantic and Northeast Pacific continental shelf seas (p. 1.3 MBytes) [Application/vnd.openxmlformats-officedocument.spreadsheetml.sheet]. PANGAEA. <https://doi.org/10.1594/PANGAEA.900866>

Froese, R. and D. Pauly. Editors. 2024.FishBase. World Wide Web electronic publication. [www.fishbase.org](http://www.fishbase.org)

Hobson, E. (1972). Activity of Hawaiian Reef Fishes During the Evening and Morning Transitions Between Daylight and Darkness: Vol. Fishery Bulletin. National Marine Fisheries Service.

Kuiter, R. H., & Tonozuka, T. (2001). Pictorial guide to Indonesian reef fishes. Part 3. Jawfishes—Sunfishes, Opistognathidae—Molidae. Zoonetics, Australia.

Lieske, E., & Myers, R. (1994). Collins Pocket Guide. Coral reef fishes. Indo-Pacific & Caribbean including the Red Sea. Haper Collins Publishers.

Mundy, B. (2005). Checklist of the fishes of the Hawaiian Archipelago (Vol. 6). Bishop Museum Pr.

Myers, R. F. (1991). Micronesian reef fishes. Second Ed. Coral Graphics, Barrigada, Guam.

Randall, J. (2010). Snorkeler’s Guide To The Fishes Of Hanauma Bay. https://repository.library.noaa.gov/view/noaa/40569

Sommer, Schneider, & Poutiers. (1996). FAO species identification field guide for fishery purposes. The living marine resources of Somalia.
